# Effectiveness of the Internet of Things for Improving Pregnancy and Postpartum Women’s Health in High-Income Countries: A Systematic Review and Meta-Analysis of Randomized Controlled Trials

**DOI:** 10.3390/healthcare13172103

**Published:** 2025-08-23

**Authors:** Etsuko Nishimura, Noyuri Yamaji, Kiriko Sasayama, Md. Obaidur Rahman, Katharina da Silva Lopes, Citra Gabriella Mamahit, Mika Ninohei, Phyu Phyu Tun, Rina Shoki, Daichi Suzuki, Aya Nitamizu, Daisuke Yoneoka, Eiko Saito, Erika Ota

**Affiliations:** 1 Faculty of Nursing, Komazawa Women’s University, Tokyo 206-8511, Japan; e-nishimura@komajo.ac.jp; 2 Institute of Clinical Epidemiology, Showa Medical University, Tokyo 142-8555, Japan; 3 Graduate School of Medicine, The University of Tokyo, Tokyo 113-0033, Japan; 4 Sustainable Society Design Center, Graduate School of Frontier Sciences, The University of Tokyo, Chiba 277-0882, Japan; 5 Department of Epidemiology, National Institute of Infectious Diseases, Japan Institute for Health Security, Tokyo 102-0071, Japan; 6 Center for Evidence-Based Medicine and Clinical Research, Dhaka 1230, Bangladesh; 7 Faculty of Health Science, University of Potsdam, 14469 Potsdam, Germany; 8 Nezu Biotech GmbH, Tiergartenstrasse 15, 69121 Heidelberg, Germany; 9 Graduate School of Nursing, St. Luke’s International University, Tokyo 104-0044, Japan; 10 School of Nursing, Dokkyo Medical University, Tochigi 321-0293, Japan; 11 Faculty of Nursing at Saitama, Japanese Red Cross College of Nursing, Saitama 338-0001, Japan; 12 Institute for Global Health Policy Research, Bureau of Global Health Cooperation, Japan Institute for Health Security, Tokyo 162-8655, Japan; 13 School of Nursing, Tokyo Medical University, Tokyo 160-8402, Japan

**Keywords:** Internet of Things, IoT, women’s health, pregnant women, postpartum women, meta-analysis, systematic review, high-income countries

## Abstract

**Background/Objectives**: The Internet of Things (IoT), integrated with application software, has increasingly been used to support health management through monitoring indicators like physical activity, sleep, and heart rate, in pregnant and postpartum women. However, limited evidence exists regarding its effectiveness in improving health outcomes for pregnant and postpartum women. The objective of this systematic review and meta-analysis was to evaluate and synthesize the role of IoT in enhancing the health outcomes of pregnant and postpartum women. **Methods**: A systematic search was conducted on 13 February 2023, across CENTRAL, CINAHL, ClinicalTrials.gov, Embase, MEDLINE, PsycINFO, PubMed, and WHO ICTRP to identify all randomized controlled trials. Studies were included if they involved pregnant or postpartum women in high-income countries and used sensor-based data collection via smartphones or wearable devices. Two reviewers independently selected the studies, extracted data, and assessed the risk of bias using the Cochrane Collaboration’s risk of bias assessment tool 2.0. We performed a pairwise meta-analysis using a random effects model. The findings were reported according to PRISMA guidelines. **Results**: Seven studies with 1638 pregnant and postpartum women were included in this review. Of the seven included studies, half targeted women with gestational diabetes and the other half targeted obese women. A meta-analysis revealed that IoT interventions may reduce gestational weight gain in women with obesity with a mean difference of −3.35 kg (95% confidence interval (CI): −5.23 to−1.46; *I*^2^ = 36%; two studies; 242 women; moderate certainty of evidence). **Conclusions**: This review suggested that IoT interventions may limit gestational weight gain in pregnant women with obesity. Future studies should evaluate the long-term effects of IoT-based interventions on maternal and neonatal health outcomes.

## 1. Introduction

Globally, the prevalence of obesity among women has been increasing since the 1970s [1]. Approximately one in five pregnant women in high-income countries is classified as obese during antenatal appointments [2]. Overweight and obesity are associated with adverse pregnancy outcomes like maternal mortality, childbirth complications, gestational diabetes, preterm birth, and intrauterine growth restriction [3,4,5]. Additionally, women with gestational diabetes have a high risk of developing type 2 diabetes mellitus within 5 years after delivery [6].

These pregnancy and postpartum complications are often associated with lifestyle behaviors such as diet, exercise, and sleep [7,8,9]. Recent studies have utilized the Internet of Things (IoT) integrated with application software to improve women’s lifestyle behaviors and health management [10,11,12]. The IoT is a technological phenomenon arising from the advancement in information and communication technologies [13]. Tsirmpas et al. defined IoT as the ability of smart devices to communicate with each other and create interconnected networks [13]. Previously, patient health and behavior-related data were limited to intermittent measurements. However, advancements in wearable sensors and systems have made real time, remote data collection and analysis of patient information feasible, facilitating applications in medical fields such as early disease detection, chronic condition prevention, and rapid emergency response [14].

Various types of IoT devices have been developed, including smartwatches, smart rings, smart clothing, and smart glasses. These devices are accompanied by a diverse array of applications aimed at fostering behavioral change, e.g., step-counting smartphone applications to improve postpartum weight among women with gestational diabetes [15,16] and smart wristbands that monitor health indicators, such as physical activity, sleep patterns, and heart rate among pregnant women [17,18].

Previous systematic reviews have examined IoT interventions targeting maternal and child health. These include reviews of wearable sensor technologies for monitoring the health of pregnant women and fetuses [19]; IoT architectures, systems, models, and devices for monitoring and managing complications during pregnancy, the postpartum period, and neonatal care [20]; and remote monitoring via digital glucose meters to aid glycemic control in women with gestational diabetes [21]. However, these studies summarized the findings narratively and did not conduct meta-analyses.

In recent years, randomized controlled trials (RCTs) have been conducted to evaluate the effectiveness of IoT interventions using wearable devices to improve the health of pregnant and postpartum women. By defining IoT interventions as sensor-driven technologies capable of tracking physiological and behavioral health indicators, it becomes feasible to synthesize evidence exclusively from RCTs. In high-income countries, the adoption of IoT technologies, such as smartphones and wearable devices, is widespread, and their integration with healthcare services and internet infrastructure is well established [22]. Therefore, this review aimed to evaluate the clinical effectiveness of IoT-based interventions on maternal and neonatal outcomes among pregnant and postpartum women in high-income countries.

## 2. Materials and Methods

### 2.1. Study Design

The systematic review protocol was registered on PROSPERO (register number: CRD42022384620) and published in a journal [23]. Initially intended as a network meta-analysis, the study was subsequently conducted as a systematic review and meta-analysis owing to the insufficient number of studies and limited variety of interventions. This study was conducted in accordance with the guidelines of the *Cochrane Handbook for Systematic Reviews of Interventions* [24]. The findings are reported (Table S1) according to the Preferred Reporting Items for Systematic Reviews and Meta-analyses (PRISMA) guidelines [25].

### 2.2. Inclusion and Exclusion Criteria

The eligibility criteria are defined in the following PICOS framework (P—Participants, I—Interventions, C—Comparator, O—Outcomes, and S—Study design):

#### 2.2.1. Participants

The study participants were pregnant or postpartum women in high-income countries. For the definition of high income, we used the World Bank classification [26].

#### 2.2.2. Intervention

Studies on IoT interventions designed to improve the health of pregnant or postpartum women were included. To evaluate the effectiveness of sensor-driven IoT applications in tracking physiological and behavioral health indicators, this review encompasses various types of IoT, with a special focus on data collection and monitoring through sensors in smartphones and wearable devices. Thus, mHealth interventions—which involve the use of mobile technologies such as mobile phones, patient monitoring tools, PDAs, and other wireless devices to facilitate the delivery of medical care and public health services [27]—and IoT interventions primarily designed for health education purposes, were excluded from the study.

#### 2.2.3. Comparators

Studies that evaluated the effectiveness of the IoT intervention compared to usual care, interventions that did not use IoT, or alternative approaches such as education or exercise without IoT, were included.

#### 2.2.4. Outcomes

Studies that reported maternal and neonatal health outcomes were included and categorized into primary and secondary outcomes. The primary outcomes were maternal and neonatal health outcomes, as they were directly related to the overall purpose of this review, which was to evaluate the effectiveness of IoT interventions in improving clinically significant health outcomes during pregnancy and the postpartum period. The secondary outcomes included lifestyle and behavioral changes, which serve as intermediate indicators that facilitate the achievement of the primary health outcomes.

Although defined as secondary outcomes, behavioral and lifestyle changes like increased physical activity, dietary improvements, and regular self-monitoring act as crucial mediators of the clinical effects of IoT interventions. These behavioral modifications represent key mechanisms through which improvements in maternal and neonatal outcomes are achieved. Therefore, evaluating these mediators is crucial to fully understand the mechanisms of impact and the effectiveness of the intervention beyond the direct clinical endpoint. This hierarchical classification aligns with the *Cochrane Handbook for Systematic Reviews of Interventions* [24], which emphasizes prioritizing clinically significant outcomes that directly impact patient care.

- Primary maternal health outcomes included health status, such as the number of cases diagnosed or treated for high-risk pregnancies (e.g., hypertensive disorders of pregnancy, gestational diabetes, and preterm delivery). Neonatal health outcomes included low birth weight, defined as birth weight <2.5 kg, and perinatal death.
- Secondary outcomes included lifestyle and behavioral changes, maintenance of a healthy weight, and other indicators such as body mass index (BMI), body composition, waist circumference, and increased physical activity.

#### 2.2.5. Study Designs

Individual and cluster RCTs were included to evaluate the effectiveness of IoT interventions on the health of pregnant and postnatal women. Reviews, qualitative studies, observational studies, cross-sectional studies, case studies, commentaries, editorials, expert opinions, and letters were excluded from this study.

### 2.3. Search Methods for Study Identification

The CENTRAL, CINAHL, ClinicalTrials.gov, Embase, MEDLINE, PsycINFO, PubMed, and WHO ICTRP databases were searched from inception to 13 February 2023, with no restrictions on date/time, language, document type, or publication status. The core search terms included combinations of keywords and MeSH terms related to “pregnancy,” “postpartum,” “Internet of Things,” “mHealth,” and “wearable devices,” with filters applied to identify RCTs. The detailed strategies are provided in Supplementary Table S2. The *Cochrane Handbook for Systematic Reviews of Interventions* [24] and Cochrane’s MECIR [28] were utilized to conduct the search. The PRISMA-S [29] and PRISMA guidelines [25] were followed for reporting the search, and the PRESS guidelines were applied for peer-reviewing the search strategies [30]. Keywords were collected through expert opinions, literature reviews, controlled vocabulary, and an examination of the primary search results (Table S2).

### 2.4. Study Selection

Duplicate records identified from the databases were excluded. Pairs of reviewers were selected from among the twelve authors (EN, NY, KS, PPT, MRO, MN, GM, KDSL, RS, DS, AN, HH) and subsequently independently screened the titles and abstracts of the records using the predefined study eligibility criteria. The full texts of the studies included in the initial screening were independently assessed by the two authors. The Rayyan tool was used during these screening processes [31]. Any disagreements between reviewers during the screening were resolved through discussion. If an agreement could not be reached, the reviewers discussed the issue with a third reviewer who made the final decision.

### 2.5. Data Extraction

The two authors used a predesigned data extraction form to extract data from the included studies. The data extraction form was designed to use Excel to extract the following information: study setting, participants, interventions, comparisons, study design, and outcome measures, including primary and secondary outcomes.

### 2.6. Risk of Bias

Two authors used the Cochrane Collaboration’s risk of bias assessment tool 2.0 to independently assess the risk of bias for each study outcome [32]. The Cochrane Collaboration’s risk of bias assessment tool 2.0 provides a framework for considering the risk of bias in the findings of any type of randomized trial. Assessments using this tool are conducted not for each trial as a whole, but for each outcome of a single trial [32]. This tool identifies the following five domains where bias may be introduced into the results: bias arising from the randomization process; bias due to deviations from intended interventions; bias due to missing outcome data; bias in measurement of the outcome; and bias in selection of the reported result [32]. Any disagreements between reviewers regarding the risk of bias were resolved through discussion. If an agreement could not be reached, the reviewers discussed the issue with a third reviewer.

### 2.7. Data Synthesis and Analytical Approach

A pairwise meta-analysis was conducted using a random-effects model to estimate the pooled effect size for each outcome combination. Given the limited number of included studies, we prioritized the use of a traditional pairwise meta-analysis to enhance the interpretability and reproducibility of the results. An intention-to-treat analysis was used to conduct the meta-analysis utilizing the Review Manager Version 5.4 software (RevMan 5). The heterogeneity among the studies included in the meta-analysis was assessed using the estimated value of *τ*^2^, which corresponds to the between-study variance, and the Chi-squared test. Additionally, graphical assessments were made using horizontal lines in the forest plots for each outcome. The *I*^2^ statistic was used to examine heterogeneity, with values ≥75% indicating considerable heterogeneity, as defined in the *Cochrane Handbook for Systematic Reviews of Interventions* [24]. Risk ratios (RRs) were used for dichotomous data, whereas mean differences (MDs) were used for continuous data, accompanied with their corresponding 95% confidence intervals (CIs). At least two studies were required as a criterion for conducting a meta-analysis. When the number of studies was less than two, the results were narratively described in accordance with the study protocol. We did not test for publication bias using funnel plots and Egger tests in the pairwise meta-analyses owing to an insufficient number of studies. Sensitivity analysis was performed if the studies had a high risk of bias.

### 2.8. Assessment of the Certainty of the Evidence

The certainty of evidence regarding the effectiveness of the IoT on outcomes for pregnant and postpartum women was assessed using the Grading of Recommendations, Assessment, Development and Evaluation (GRADE) approach [33]. This system assesses the certainty of evidence and grades it into four categories: high, moderate, low, and very low [33]. The certainty of evidence may be downgraded if there is an increased risk of bias in the studies, inconsistency of results across studies, indirectness of evidence, imprecision of estimates, or concerns about publication bias [34]. In accordance with the protocol, a table title “Summary of findings” was generated using GRADEPro [35] (https://www.gradepro.org/product, accessed on 30 April 2025) for the primary outcome results (Table S3).

## 3. Results

### 3.1. Search Results

**Figure 1:**
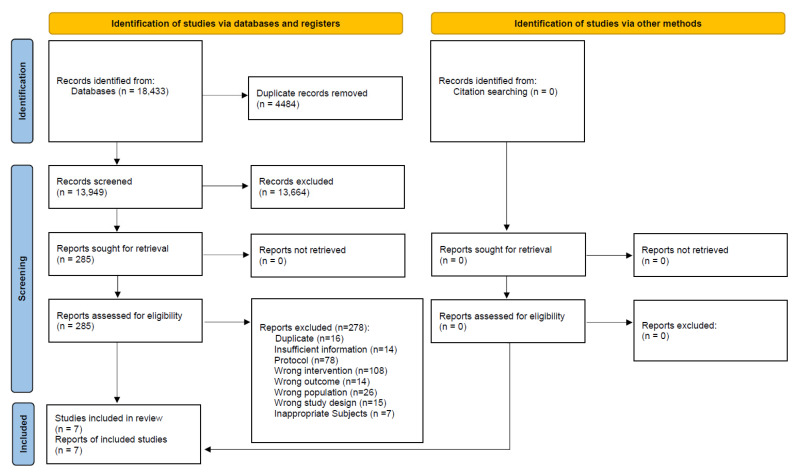
Flow diagram of the search results and study selection for pregnant and postpartum women.

A total of 18,433 records were identified from all targeted electronic databases and other resources, excluding 4484 duplicate records. Moreover, 278 reports were excluded during full-text screening: 141 for ineligible population or intervention, 15 for ineligible study design, 78 study protocols, 16 duplicates, 14 for ineligible outcomes, and 14 for insufficient information. Finally, seven studies were included in this review. The study selection process is described in the flow diagram (Figure 1).

### 3.2. Characteristics of the Included Studies

All seven studies included in this review were individual RCTs, half of which were pilot RCTs. These studies were conducted in the United States, Australia, South Korea, Taiwan, Spain, Singapore, and Belgium. Overall, 1638 pregnant and postpartum women participated in the studies. Of the seven included studies, half targeted women with gestational diabetes [15,36,37] and the other half targeted women with obesity [38,39,40,41]. All interventions included activity trackers or wearable devices to monitor physical activity and/or body weight in real time. In addition, all interventions incorporated feedback and counseling provided to the study participants. The characteristics of the included studies are detailed in Table 1 and Tables S4–S8 in the Supplementary Materials. In the included studies, no significant missing data and outliers were identified for key primary and secondary outcomes. Ethics approval was obtained from the relevant institutional review boards in all seven trials; however, the IRB numbers were not listed in five studies.

### 3.3. Overall Risk of Bias Assessment of the Included Studies

**Figure 2:**
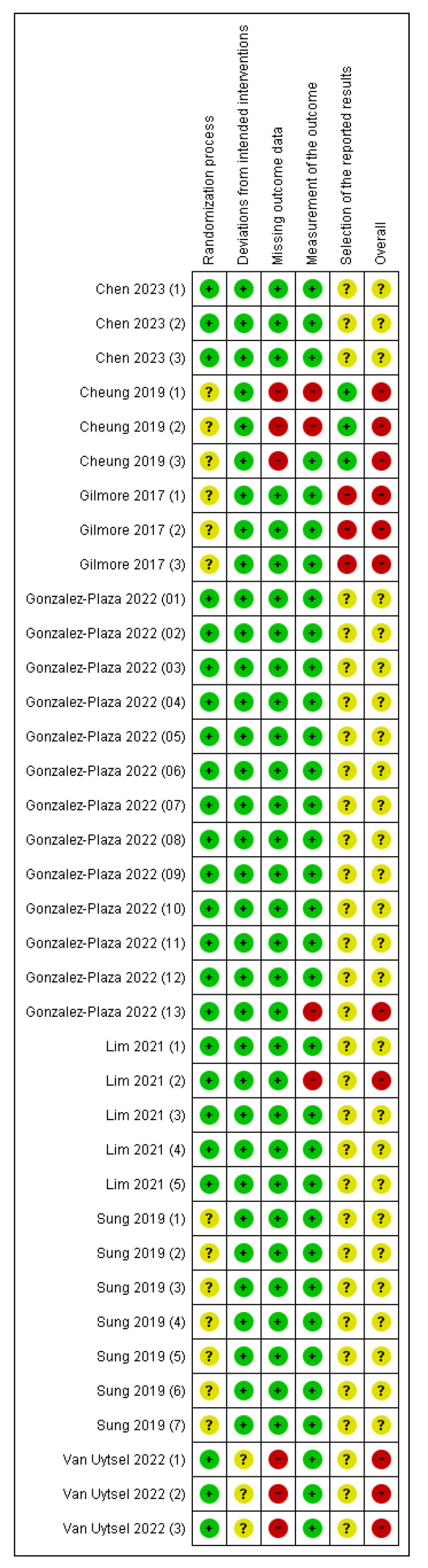
Risk of bias summary [15,36,37,38,39,40,41]. Red with a - symbol: high risk of bias, yellow with a ? symbol: unclear risk of bias, and green with a + symbol: low risk of bias. Chen 2023(1): Gestational weight gain (kg) first trimester, Chen 2023(2): Gestational weight gain (kg) second trimester, Chen 2023(3): Gestational weight gain (kg) third trimester, Cheung 2019(1): Weight change between final evaluation and 10–12 weeks post-partum (kg), Cheung 2019(2): Total weekly activity time (minutes) at final evaluation, Cheung 2019(3): 7-day pedometer step count, Gilmore 2017(1): Change in weight, Gilmore 2017(2): Body fat percentage, Gilmore 2017(3): Waist circumference, Gonzalez-Plaza 2022(1): Unplanned cesarean, Gonzalez-Plaza 2022(2): Gestational weight gain (kg), Gonzalez-Plaza 2022(3): Gestational weight gain–weekly weight gain (kg), Gonzalez-Plaza 2022(4): Composite pregnancy morbidity, Gonzalez-Plaza 2022(5): Miscarriage ≤22 weeks, Gonzalez-Plaza 2022(6): Gestational diabetes, Gonzalez-Plaza 2022(7): Preeclampsia or gestational hypertension, Gonzalez-Plaza 2022(8): Preterm labor ≤37 weeks, Gonzalez-Plaza 2022(9): Birthweight ≤2500 g, Gonzalez-Plaza 2022(10): Perinatal death, Gonzalez-Plaza 2022(11): Early neonatal death, Gonzalez-Plaza 2022(12): Antepartum stillbirth, Gonzalez-Plaza 2022(13): Physical activity (moderate to high), Lim 2021(1): Absolute difference in weight (kg) from first trimester, Lim 2021(2): Waist circumference (cm), Lim 2021(3): Systolic blood pressure (mm Hg), Lim 2021(4): Fasting OGTT test (mmol/L), Lim 2021(5): 2 h OGTT (mmol/L), Sung 2019(1): Cesarean section, Sung 2019(2): Weight gain during study period (kg), Sung 2019(3): Body fat percentage at postpartum, Sung 2019(4): Systolic blood pressure (mm Hg), Sung 2019(5): Fasting glucose, mg/dL, Sung 2019(6): HOMA-IR, Sung 2019(7): Diabetes at 4–12 weeks postpartum, Van Uytsel 2022(1): Weight retention (kg) change after intervention (6 months postpartum), Van Uytsel 2022(2): Body fat percentage change after intervention (6 months postpartum), Van Uytsel 2022(3): Waist circumference (cm) change after intervention (6 months postpartum).

The most frequently identified biases across outcomes were selection bias (risk of bias arising from the randomization process) and reporting bias (risk of bias in the selection of the reported result) (Figure 2 and Figure S1). Most outcomes regarding reporting bias were unclear. Therefore, the “overall” risk of all outcomes was either unknown or high. The blinding of participants and assessors was not feasible due to the nature of the interventions. However, deviations from the intended interventions were unlikely due to the implementation context of the trials. Furthermore, most outcomes assessed using the risk of bias tool relied on objective data, indicating that the potential impact of the lack of blinding on bias was minimal. The studies conducted in Australia [36] and Belgium [41] had high dropout rates, the reasons for which were unclear, leading to a high risk of bias in missing outcome data (Figure 2). In the study conducted in Spain [40], some participants were excluded due to strict COVID-19 lockdown measures; however, the reasons were documented and unrelated to the intervention, resulting in a low risk of bias in missing outcome data. Despite the use of RCTs, the overall risk of bias was high, particularly due to the attrition and outcome reporting domains.

### 3.4. Effects of IoT Interventions on Improving Pregnant and Postpartum Women’s Health Outcomes

**Figure 3:**
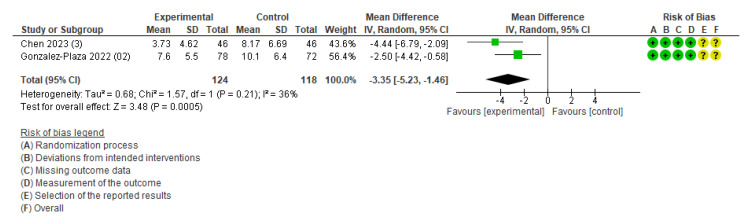
Meta-analysis for the effect of IoT interventions vs. no IoT intervention on gestational weight gain (kg) [38,40]. Yellow with a ? symbol: unclear risk of bias, and green with a + symbol: low risk of bias.

**Figure 4:**
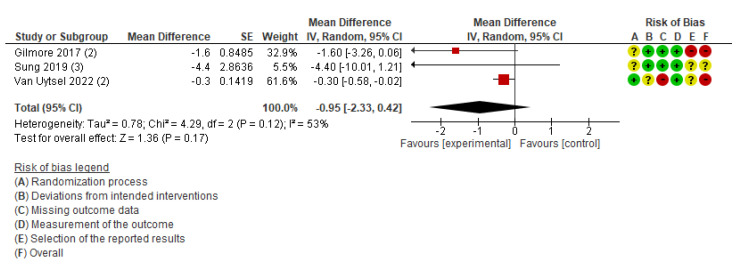
Meta-analysis for the effect of IoT interventions vs. no IoT intervention on fat change (%) of postpartum women [37,39,41]. Red with a - symbol: high risk of bias, yellow with a ? symbol: unclear risk of bias, and green with a + symbol: low risk of bias.

The primary outcomes of maternal health (cesarean section, pregnancy complications, and postpartum diabetes) and neonatal health (low birth weight, perinatal death, neonatal death, and stillbirth) were not available for the meta-analysis owing to an insufficient number of studies. The meta-analysis was performed for three outcomes: weight change (Figure S2), gestational weight gain (Figure 3), and body fat (Figure 4). Table S3 demonstrates the certainty of evidence for the effectiveness of the IoT on the outcomes, which was assessed using the GRADE approach. The certainty was rated as moderate for GWG and low to very low for all other outcomes.

#### 3.4.1. Maternal Health Outcomes

One study of pregnant women with a BMI of ≥30 kg/m^2^ reported unplanned cesarean sections, with no difference between the intervention and control groups (RR, 1.38; 95% CI: 0.60 to 3.19; 150 pregnant women) [40]. No differences were observed in the prevalence of composite pregnancy morbidity (RR, 0.85; 95% CI: 0.52 to 1.37; 150 pregnant women), preeclampsia or gestational hypertension (RR, 0.62; 95% CI: 0.23 to 1.64; 150 pregnant women), gestational diabetes (RR, 0.77; 95% CI: 0.35 to 1.67), miscarriage ≤22 weeks (RR, 4.62; 95% CI: 0.23 to 94.64; 150 pregnant women), and preterm labor ≤37 weeks (RR, 1.29; 95% CI: 0.43 to 3.89; 150 pregnant women) between the intervention and control groups [40].

Similarly, a study of women with gestational diabetes in South Korea reported no difference in the prevalence of diabetes at postpartum between the intervention and control groups (RR, 0.91; 95% CI: 0.16–5.30; 21 pregnant women) [37].

Although maternal clinical outcomes such as cesarean section, gestational hypertension, and postpartum diabetes were reported, none of the results reached statistical significance. However, these important clinical outcomes were assessed in a small number of studies, limiting the feasibility of conducting a meta-analysis and reducing the generalizability of the findings.

#### 3.4.2. Neonatal Health Outcomes

Regarding neonatal health outcomes, the study conducted in Spain reported no difference in the prevalence of birthweight ≤2500 g (RR, 2.31; 95% CI: 0.46 to 11.52; 150 pregnant women), perinatal death (RR, 0.46; 95% CI: 0.04 to 4.98), early neonatal death (RR, 0.92; 95% CI: 0.06 to 14.49), and antepartum stillbirth (RR, 0.31; 95% CI: 0.01 to 7.44) between the intervention and control groups [40]. Owing to the limited number of studies and wide CIs, no firm conclusions could be drawn regarding the neonatal benefits of IoT-based interventions.

#### 3.4.3. Maintaining a Healthy Weight or Other Indicators

We conducted the meta-analysis to synthesize the intervention effect of the outcome of mean weight change at postpartum. This outcome included three studies [36,39,41]. The pooled estimates showed no difference between the intervention and control groups (MD, −0.25; 95% CI: −0.62 to 0.12, *I*^2^ = 0%, three studies, 1550 women, very low certainty of evidence) (Figure S2). According to the GRADE assessment, the certainty of evidence for this outcome was very low. In addition, as two of the three studies were pilot trials with small sample sizes, the analysis may have been underpowered to detect a statistically significant difference.

Other weight-related outcomes included gestational weight gain and optimal weight at four months postpartum. The outcome of gestational weight gain included two studies [38,40]. The study conducted in Taiwan was an RCT of pregnant women with a BMI ≥ 25 kg/m^2^, and participants in the intervention group were required to achieve 8500 steps per day and wear the smartband Mi Band 5 (WAT) [38]. Another study conducted in Spain also targeted pregnant women with obesity (BMI > 30 kg/m^2^), and participants in the intervention group were advised to walk 10,000 steps per day and wear a smartband (Mi Band 2) synchronized with an application offering health counseling and midwifery support [40]. The pooled estimates showed that gestational weight gain was 3.35 kg lower in the intervention compared to that in the control group (MD, −3.35; 95% CI: −5.23 to −1.46; *I*^2^ = 36%; two studies; 242 women; moderate certainty of evidence) (Figure 3). The outcome of the number of women achieving optimal weight at four months postpartum included only one study [40], and the intervention group did not significantly differ from the control group.

This outcome included three studies conducted in the USA [39], South Korea [37], and Belgium [41]. The participants in these studies were postpartum women with a BMI of 25–40 kg/m^2^ [39], women with excessive gestational weight gain [41], and women diagnosed with gestational diabetes mellitus [37]. The percentage of body fat was measured postpartum in all three studies. Using a fixed-effects model for meta-analysis, the results indicated moderate heterogeneity (MD, −0.35; 95% CI: −0.62 to −0.07; *I*^2^ = 53%). Therefore, a sensitivity analysis was conducted using a random-effects model, which showed no statistically significant difference between the intervention and comparison groups (MD, −0.95; 95% CI: −2.33 to 0.42; *I*^2^ = 53%; three studies; 1,511 women; very low certainty of evidence) (Figure 4). The difference in results between the fixed-effects and random-effects models may reflect methodological heterogeneity, such as variations in the types of devices used (e.g., accelerometers, wearable activity trackers), study settings, and participant characteristics.

One study conducted in Belgium [41] reported the outcome of waist circumference and found no difference between the intervention and control groups (RR, −0.10; 95% CI: −0.57 to 0.37; 1450 women).

#### 3.4.4. Physical Activity

Three studies reported physical activity [36,40]. However, a meta-analysis could not be conducted since each study used different ways of measuring physical activity. The findings for physical activity were as follows: the 7-day pedometer step count (MD, 6600.00; 95% CI: −4945.03 to 18,145.03; 60 women) [36], total weekly activity time (min) at final evaluation (MD, 67.00; 95% CI: −32.95 to 166.95; 60 women) [36], and physical activity (moderate to high) (RR, 1.25; 95% CI: 0.97 to 1.63; 150 pregnant women) [40]. The lack of standardization in physical activity measurement tools (e.g., self-reports vs. accelerometers) restricted comparability across studies.

## 4. Discussion

### 4.1. Principal Findings

In this systematic review, we evaluated the effectiveness of IoT interventions on the health of pregnant and postpartum women. The meta-analysis indicated that IoT interventions might help reduce gestational weight gain among obese pregnant women; however, this conclusion was based on only two RCTs involving a combined sample of 242 women. The limited number of studies and relatively small sample size indicate that the evidence is preliminary and should be interpreted with caution. Moreover, the meta-analysis revealed moderate heterogeneity (*I*^2^ = 36 %), suggesting that differences in study design or interventions may have influenced the estimated effect.

Therefore, while the potential effect of IoT interventions is clinically promising, the certainty of evidence remains moderate, and confirmation in larger, well-designed trials is warranted.

Two studies [38,40] that focused on preventing excessive gestational weight gain in obese pregnant women aimed to manage weight gain within the guidelines set by the Institute of Medicine [42]. The IoT-based interventions led to a 3.35 kg reduction in gestational weight gain compared to the control groups. Higher pre-pregnancy BMI and excessive gestational weight gain are associated with an increased risk of adverse maternal and neonatal outcomes [43,44], including gestational hypertension, gestational diabetes, and large-for-gestational-age infants [44]. Systematic reviews and meta-analyses on gestational weight gain among women with obesity have reported that restricting gestational weight gain reduces the risk of LGA [45,46], lowers the likelihood of cesarean delivery [45,46], and may also decrease the incidence of hypertensive disorders such as preeclampsia [46]. Therefore, the reduction in gestational weight gain observed among obese pregnant women through IoT-based interventions may have clinically important implications.

The interventions employed behavior-change strategies based on Bandura’s Social Cognitive Theory (SCT) [47,48], focusing on both diet and physical activity. In the study conducted in Taiwan, nurses supported pregnant women’s health behaviors through personalized SMS messages [38], and in the study conducted in Spain, midwives provided individualized health information and responded to questions via a mobile application [40]. From an IoT perspective, both studies utilized wearable activity monitors (smartbands) and mobile applications to track physical activity [38,40]. In health promotion practices, components of SCT are widely used and have been suggested to positively influence health outcomes and intervention effectiveness [49]. Within SCT, the belief in self-efficacy plays a crucial role, as enhancing self-efficacy directly or indirectly leads to behavioral change [48]. Self-monitoring and self-management are key factors in increasing self-efficacy, and the IoT interventions facilitate this process by enabling individuals to track their physical activity and manage their health more effectively. Differences in professional support (nurses versus midwives), cultural context, and intervention intensity across these two trials may partly explain the moderate heterogeneity observed in our meta-analysis. These contextual variations highlight the need for caution when generalizing the findings and underscore the importance of future research with more standardized interventions. Although the included interventions ranged from simple step counters to more complex coaching programs, none of the trials delivered a wearable-only intervention; all combined wearable devices with some form of counseling or coaching. Therefore, subgroup analyses comparing wearable-only versus wearable-plus-coaching interventions were not feasible, and the conceptual heterogeneity of the interventions should be kept in mind when interpreting the pooled results.

A Cochrane review published in 2015 indicated that interventions targeting diet, exercise, or both during pregnancy may mitigate the risk of excessive gestational weight gain [50]. While the Cochrane review did not conduct a meta-analysis based on BMI classification [50], women with high BMI values in early pregnancy tend to have lower activity levels and are more prone to increased gestational weight gain [51]. The current study suggests that IoT interventions utilizing smartbands may contribute to increased physical activity levels and reduced gestational weight gain in pregnant women with obesity. However, given the modest sample size, limited number of studies, and moderate heterogeneity in the meta-analysis, the magnitude and generalizability of this effect remain uncertain.

Only a limited number of studies reported key clinical outcomes, such as cesarean section, gestational diabetes, and neonatal morbidity; existing evidence is insufficient to draw firm conclusions regarding the clinical effectiveness of IoT interventions. This gap underscores the need for future high-quality studies that standardize the reporting of clinically important maternal and neonatal outcomes.

The results of the present meta-analysis did not show any significant effect of IoT interventions on postpartum weight reduction. This outcome encompassed three studies [36,39,41]. As an intervention, a wearable activity monitor and a mobile application were used to provide dietary and physical activity interventions [36,39,41]. Combined dietary and exercise interventions have been considered optimal strategies for promoting postpartum weight loss [52]. However, previous studies have reported inconsistent findings regarding the success of postpartum weight reduction [53]. In our meta-analysis, the lack of significant improvement in postpartum weight may be attributed to several contextual and methodological factors. One possible reason is the relatively short duration of interventions in the included studies, which ranged from four to six months. This period may have been insufficient to observe substantial behavioral changes and the resulting impact on body weight [41]. Moreover, two studies identified high dropout rates and low adherence to activity tracker usage as key challenges [36,41]. Possible reasons included postpartum lifestyle changes and technical difficulties. A study conducted in Australia reported that primiparous women and South Asian women were significantly more likely to not complete the study [36]. Additionally, two of the three included studies were pilot trials [36,39], and their small sample sizes may have resulted in insufficient statistical power, potentially obscuring the true effects of the intervention.

Postpartum body fat is a crucial outcome since a high body fat percentage is associated with increased health risks, even if the BMI is within the normal range [54,55].The interventions targeted women with postpartum overweight or obesity [39], those with excessive gestational weight gain [41], and those with gestational diabetes [37], utilizing interventions involving Bluetooth-connected activity trackers and scales [39,41] or monitoring system devices, including Bluetooth-connected glucometers and accelerometers for detecting physical activity levels [37]. When the meta-analysis was performed using a fixed-effects model according to the protocol, women in the IoT intervention group experienced a slight reduction in body fat percentage. However, after switching to a random-effects model to account for heterogeneity in intervention methods and study populations, the statistical significance disappeared, and the robustness of the effect was not demonstrated [37,39,41]. One possible reason for the lack of significant differences between the intervention and comparison groups is the short duration of the intervention. Postpartum women who are overweight or obese tend to experience slower reductions in body weight and fat percentage compared to those with normal weight, suggesting that longer-term interventions may be necessary.

Additionally, two of the three studies were pilot trials [37,39], and their small sample sizes may have limited the statistical power to detect significant effects. The inconsistency between the fixed effects and random effects results suggests that the pooled effect is sensitive to assumptions about between-study variance. Potential sources of heterogeneity include differences in device type (e.g., Bluetooth-connected scales versus comprehensive monitoring systems), variation in participant populations (postpartum women with overweight/obesity, excessive gestational weight gain, or gestational diabetes), and the length of follow-up across trials. Moreover, the total number of participants contributing to the body fat outcome was small, implying that the analyses may have been underpowered to detect modest but clinically meaningful effects.

Furthermore, waist circumference is an important indicator as it is associated with the incidence and mortality of future cardiometabolic diseases [56,57]. Regarding postpartum women, changes in weight and waist circumference in those who developed GDM have been suggested to influence cardiometabolic risk factors even in the first year postpartum [58]. However, due to difficulties in comparing results across studies, a meta-analysis could not be conducted.

A meta-analysis on physical activity could not be performed due to heterogeneity in measurement methods across the included studies. The lack of standardization in physical activity measurement tools (e.g., self-report vs. accelerometers, varying definitions of moderate activity) limits cross-study comparability and highlights the need for harmonized metrics in IoT-based intervention studies.

In the current review, the application of RoB 2.0 revealed that many of the included trials were rated as having “some concerns” or “high risk” due to the lack of participant blinding, high attrition bias, and incomplete outcome reporting. Moreover, most studies were small pilot trials with short follow-up periods; therefore, the GRADE assessments rated the certainty of evidence as low to moderate for gestational weight gain and as low certainty for postpartum outcomes, such as weight retention and body fat percentage. These methodological limitations may decrease the confidence in the observed effects and indicate the need for larger, high-quality trials. Several of the included trials exhibited a high risk of bias or “some concerns” in the domains of randomization and selective outcome reporting. Inadequate randomization can introduce baseline imbalances, and selective reporting may distort effect estimates, both of which threaten the internal validity of the findings. For example, Cheung et al. 2019 and Van Uytsel et al. 2022 had high dropout rates leading to missing outcome data [36,41], while Chen et al. 2023 had unclear allocation concealment [38]; these methodological issues raise concerns about bias. We had planned to conduct sensitivity analyses excluding high-risk trials, but only two studies contributed to the gestational weight-gain meta-analysis, and the postpartum analyses were based on three small pilot trials; therefore, conducting a meta-analysis excluding these studies was not feasible. Consequently, the pooled results should be interpreted with caution, and future research should prioritize robust randomization procedures and complete reporting of all study details.

### 4.2. Role of IoT Interventions in Behavior Change Strategies

In health promotion practices, the components of SCT are widely utilized and are considered to have a positive impact on health outcomes and intervention effectiveness [49]. SCT identifies key determinants of behavior change, including knowledge about health risks and the benefits of health practices, self-efficacy, outcome expectations, health goals and planning, and environmental factors (facilitators and barriers). Among these, the belief in self-efficacy plays a particularly crucial role [48]. By leveraging IoT technologies such as wearable activity monitors (smartbands) and mobile applications, patients can gain health-related knowledge, visually monitor their physical activity, enhance their sense of control, set and achieve goals, and ultimately increase their health awareness [38,40]. Interventions targeting weight loss in adults with obesity often involve dietary and physical activity modifications [59]. Goal setting and self-monitoring using IoT technology are considered effective behavioral change techniques for individuals with obesity [60].

However, individuals with obesity often exhibit low adherence to interventions [61], and in studies targeting postpartum women—whose lifestyles shift due to childcare and returning to work—discontinuation due to forgetting or losing wearable devices is a common challenge [39,51]. Although there are cost considerations and physical limitations, wearable devices that do not need to be removed during daily activities or can be inconspicuously worn may be more suitable for study participants given their potential to enhance adherence and comfort.

Technical issues [36], decreased usage of IoT devices and applications over long periods [15], and cultural and social challenges [36] have also been reported. While IoT devices can function as facilitators within environmental factors, they may also act as barriers. Thus, when designing intervention methods, it is essential to consider measures to reduce barriers, such as by providing human support.

In this review, behavioral and lifestyle modifications were defined as secondary outcomes; however, changes such as increased physical activity, dietary improvements, and regular self-monitoring serve as important mediators of the clinical effects of IoT interventions. These behavioral modifications represent key mechanisms through which improvements in maternal and neonatal outcomes are achieved and are essential for understanding the pathways through which the interventions exert their effects.

### 4.3. Future Research Utilizing IoT Technology

Postpartum women experience significant lifestyle changes due to childbirth. Since RCTs using IoT technology during pregnancy have demonstrated intervention effectiveness, it is necessary to consider long-term interventions starting from pregnancy rather than from the postpartum period. Pregnancy is a time when individuals can more easily receive social support, such as support from professionals and pregnancy communities [62], making it easier to establish an environment that promotes healthy behaviors.

The studies included in this review used IoT technology primarily to track physical activity. However, future studies should also explore the possibility of integrating physiological data such as blood pressure, heart rate, and sleep, as well as physical activity levels. IoT technology is already being used for remote monitoring of hypertensive patients [63]. By utilizing multiple IoT devices to collect and analyze these data, IoT-based interventions could be adapted and developed for high-risk pregnant women, such as those with gestational hypertension. Monitoring high-risk pregnant women using IoT technologies such as wearable devices requires close integration with healthcare services [64]. Previous studies have reported that adopting a cloud-based support model enables efficient processing and storage of large volumes of data, as well as effective communication with applications, thereby addressing various aspects of utilizing data effectively [64]. This approach has the potential to contribute to continuous monitoring, early detection of abnormalities, and improved personalized management.

While this systematic review and meta-analysis focused on evaluating the effectiveness of IoT interventions in improving maternal health outcomes, future research should also consider the cost-effectiveness, scalability, and real-world implementation of these interventions. Understanding the financial feasibility and integration of IoT technologies into existing healthcare systems will be essential for their broader adoption and long-term sustainability.

From an ethical perspective, studies utilizing IoT technology must consider the possibility of a digital divide even in high-income countries. IoT interventions assume access to smartphones and wearable devices; however, these technologies may not be equally available across all socioeconomic groups. A large-scale national survey conducted among Japanese women revealed that nearly 80% of respondents had never used IoT or applications [65]. The digital divide is typically categorized into a first-order divide, which represents inequalities in physical access to digital resources, and a second-order divide, which pertains to disparities in usage [66]. While infrastructure and economic development are believed to have reduced access inequalities in developed environments [66], wearable devices may still contribute to a first-order digital divide even in high-income countries.

A study conducted in the United States found that 95.3% (4686/4915) of individuals who do not use wearable devices stated that they would use them if provided for free [67], indicating the presence of economic barriers. Additionally, prior literature on the ethical issues arising from research using IoT devices has pointed out that the widespread use of IoT devices may raise concerns regarding privacy and data security [68]. As a recommendation, it has been suggested that specific protocols should be developed to ensure both proper consent and data protection [68].

As mentioned earlier, in RCTs utilizing IoT technology, issues such as dropout rates and forgetting to wear the devices have been identified as challenges for conducting long-term research. Future studies will require interventions that address these challenges in IoT-based research. Moreover, theory-based interventions have been implemented in medical interventions [69,70] and in studies utilizing IoT technology. Theories such as SCT [38,40] and the Transtheoretical Model of Behavior Change [41] have been used to promote behavioral change, though only in a limited number of studies. It is necessary to develop interventions that can mitigate inhibitory factors as a whole and conduct evaluations that will contribute to future research.

### 4.4. Strengths and Limitations of This Review

A key strength of this review is that it is one of the most comprehensive and up-to-date reviews and meta-analyses evaluating the effectiveness of various forms of IoT in enhancing the health outcomes of pregnant and postpartum women. Interventions utilizing IoT contributed to the mitigation of gestational weight gain in pregnant women with obesity. With the increasing prevalence of maternal obesity and gestational diabetes as a public health concern [71,72], IoT-based interventions may have potential utility as a public health strategy to improve maternal and child health.

However, this study has some limitations. First, the synthesis through meta-analysis for primary outcomes was challenging owing to the scarcity of outcomes encompassing two or more studies. Additionally, many were pilot studies with small sample sizes, and some had high dropout rates, which reduced the certainty of evidence. Due to the small number of studies included and the moderate heterogeneity, this meta-analysis may not have sufficient statistical power to detect clinically meaningful differences. Another limitation is that in interventions using IoT, blinding of participants and intervention providers is impossible, which may lead to performance bias. However, the outcomes used in the meta-analysis were based on objective data in most studies, making them less susceptible to bias even if blinding was not implemented. Furthermore, this review was limited to high-income countries, which restricted the generalizability of the findings to low- and middle-income countries. Therefore, future systematic reviews should include studies conducted in low- and middle-income countries to evaluate the effectiveness of IoT-based interventions in settings where the adoption of IoT technologies and healthcare systems differ. Finally, the inclusion of interventions beyond IoT utilization, such as lifestyle coaching sessions and mobile healthcare services, alongside data collection and monitoring through sensors in wearable devices, causes difficulty in attributing the effects solely to the use of IoT. Nevertheless, a commonality across all included studies was that only the intervention groups used IoT devices to self-monitor their physical activity.

## 5. Conclusions

This review identified a moderate reduction in gestational weight gain, indicating that IoT-based interventions can be a promising tool for improving maternal weight related outcomes. Evidence from previous studies suggests that restricting gestational weight gain in obese pregnant women may reduce the likelihood of gestational diabetes, preeclampsia, and large-for-gestational-age births, contributing to better maternal and neonatal health outcomes. However, clinical outcomes such as cesarean section or neonatal morbidity were inconsistently reported and showed no significant effects, limiting the clinical generalizability of the current findings. To enhance their effectiveness, IoT-based interventions should be designed using behavior change theories and incorporate continuous monitoring of physical activity and physiological data, along with personalized, real-time feedback. Additionally, the long-term effects on maternal and neonatal outcomes should be evaluated using rigorous study designs, large sample sizes, and extended follow-up periods.

## Figures and Tables

**Table 1:** Characteristics of the included studies.

Author, Year	N (Intervention, Comparison)	Intervention	Outcomes Reported
Gilmore et al., 2017 [39]	40 (20 vs. 20)	A smartphone-based remote intervention using the SmartLoss^®^ application The IoT devices included a Bluetooth-enabled scale (BodyTrace) and a Fitbit Zip for step tracking. Personalized goals were set for both body weight and step count. Educational content covering nutrition, physical activity, and behavior change was delivered through the application. When the application-linked weight data deviated from the target zone for more than three consecutive days, an automatic trigger was activated, prompting individualized feedback from a registered dietitian via phone or email.	Primary: change in weight (kg)
Cheung et al., 2019 [36]	60 (40, 20)	A text messaging intervention program was linked to a Fitbit Flex^®^ active monitor that enabled tracking of activity and further customization of text messages that were sent during two 30 min lifestyle counseling sessions (a face-to-face session and a session by phone). Based on the step count data, adaptive weekly step goals and encouraging messages were automatically delivered.	(i) Attendance for the postpartum GTT within 12 weeks postpartum; (ii) Adherence to physical activity recommendations, constituting 30 min of moderate intensity physical activity at least 5 days a week as a self-reported outcome, along with achieving a daily step count of 10,000 recorded by pedometer count, assessed at the 6-month mark; (iii) Achievement of dietary macronutrient recommendations regarding fat and fiber intake, including a dietary fat intake of ≤30% of total daily caloric intake, saturated fat consumption below 10%, and the consumption of 15 g of fiber per 1000 calories, evaluated at the 6-month interval; (iv) Evaluation of the change in self-reported weight (kg) recorded at the 6-month follow-up.
Sung et al., 2019 [37]	21 (11, 10)	Tailored mobile health care services provided by the mobile phone application designed for the study. The IoT devices included a Bluetooth-enabled glucometer and an accelerometer for monitoring physical activity. A multidisciplinary health care team (including endocrinologists, nurses, and dietitians) reviewed the transmitted data twice weekly and provided personalized feedback and guidance through the application’s messaging system. Educational content and recommendations on diet and physical activity were also delivered regularly via the application.	Obstetrical outcomes: GA at delivery, birth weight (kg), small for GA, large for GA, cesarean section. Metabolic outcomes: maternal BMI, weight (kg), body fat (%), HOMA-IR.
Chen et al., 2023 [38]	92 (46, 46)	Nurse-led mobile health intervention. Multifunctional application design based on the behavior change theory (social cognitive theory). The IoT device used was a wrist-worn activity tracker (Mi Band 5). Individually tailored SMS text messages were delivered to promote behavioral changes in pregnant women, including encouragement based on their progress in weight management. The application provided automated feedback and included daily step goals.	Primary: rate of excessive weekly GWG (kg/week), rate of excessive total GWG (kg), changes and trajectories of GWG (kg) in both groups throughout pregnancy
Gonzalez-Plaza et al., 2022 [40]	150 (78, 72)	A complex intervention based on social cognitive theory (SCT), combining a mobile health approach using a smartband (Mi Band 2) and smartphone application (Mi Fit) with remote midwife counseling. The intervention aimed to enhance self-monitoring, self-efficacy, and outcome expectations. Through the Hangouts application, individually tailored educational messages (videos and texts) were delivered twice a week according to the gestational week. Additionally, monthly personalized feedback from a midwife and on-demand support with responses within 1 h were provided.	(i) Primary: GWG (gestation weight gain (kg)) and total physical activity.
Lim et al., 2021 [15]	200 (101, 99)	The intervention was a smartphone-based lifestyle program using the locally developed nBuddy application, designed for women with recent gestational diabetes mellitus (GDM). Calorie and activity level goals were individually tailored to achieve the target weight based on each participant’s profile. Live interaction with a research team consisting of dietitians, physiotherapists, and occupational therapists was available.	Primary: the percentage of women who regained their first trimester weight by 4 months postpartum if their first trimester BMI was ≤23 kg/m^2^, or achieved a weight loss of at least 5% from their first trimester weight if their first trimester BMI was >23 kg/m^2^.
Van Uytsel et al., 2022 [41]	1075 (551, 524)	Four face-to-face lifestyle coaching sessions using a smartphone application. A Bluetooth connection was set up with an activity tracker (Withings Go) and a weighing scale (Withings Body+). Four coaching sessions were conducted in person (at 6, 8, 12 weeks, and 6 months postpartum), focusing on nutrition, physical activity, and mental well-being. The sessions employed motivational interviewing techniques and behavior change strategies such as goal setting, action planning, and self-monitoring. The application provided continuous support between sessions, including self-tracking, goal visualization, and personalized motivational messages based on the collected data.	Weight retention (kg), fat percentage, waist and hip circumference (cm), energy intake, improved physical activity (an increase of 700 MET-minutes/week), and improved sedentary time (a decrease of 1 sedentary hours/day).

Notes: BMI, body mass index; GA, gestational age; GDM, gestational diabetes mellitus; GWG, gestational weight gain; HOMA-IR, Homeostatic Model Assessment for Insulin Resistance.

## Data Availability

Dataset available upon request from the corresponding author.

## Supplementary Materials

The following supporting information can be downloaded at: https://www.mdpi.com/article/10.3390/healthcare13172103/s1, Table S1: PRISMA 2020 checklist; Table S2: Search strategy; Table S3: Summary of findings; Table S4: Additional characteristics of the included studies; Table S5: Body fat change; Table S6: Gestational weight gain; Table S7: Weight change in postpartum women; Table S8: Summary of outcomes reported in included studies, categorized as primary clinical outcomes and behavioral/intermediate outcomes; Figure S1: Risk of bias graph; Figure S2: Meta-analysis of the effect of IoT interventions vs. no IoT intervention on the mean change in weight (kg) of postpartum women. Reference [73] is cited in the supplementary materials.

## Abbreviations

The following abbreviations are used in this manuscript:

IoT	Internet of Things
SCT	Social Cognitive Theory
BMI	body mass index
GA	gestational age
GDM	gestational diabetes mellitus
GWG	gestational weight gain
HbA1C	glycated hemoglobin
HOMA-IR	Homeostatic Model Assessment for Insulin Resistance
OGTT	oral glucose tolerance test
