# Supplementary material for: Effectiveness of the Internet of Things for Improving Pregnancy and Postpartum Women’s Health in High-Income Countries: A Systematic Review and Meta-Analysis of Randomized Controlled Trials
Source: Healthcare (Basel). 2025 Aug 23;13(17):2103. doi: 10.3390/healthcare13172103 (PMC12428080; doi:10.3390/healthcare13172103)
Supplement: Supplementary file 1 [file healthcare-13-02103-s001.zip › Figure S2. Meta-analysis_rev.pdf]

Figure S2. Meta-analysis of the effect of IoT interventions vs. no IoT intervention on the mean change in weight (kg) of postpartum women.

Study or Subgroup	Experimental Mean	Experimental SD	Experimental Total	Control Mean	Control SD	Control Total	Weight	Mean Difference IV, Random, 95% CI	A	B	C	D	E	F
Cheung 2019 (1)	-1.7	4.1	40	-1.1	3.3	20	3.7%	-0.60 [-2.53, 1.33]	?	+	-	-	+	-
Gilmore 2017 (1)	-0.1	4.0249	20	1.8	4.0249	20	2.2%	-1.90 [-4.39, 0.59]	?	+	+	+	-	-
Van Uytsel 2022 (1)	-2.6	4	724	-2.4	3.4	726	94.1%	-0.20 [-0.58, 0.18]	+	?	-	+	?	-
**Total (95% CI)**			**784**			**766**	**100.0%**	**-0.25 [-0.62, 0.12]**						

Heterogeneity: Tau² = 0.00; Chi² = 1.87, df = 2 (P = 0.39); I² = 0%
Test for overall effect: Z = 1.33 (P = 0.18)

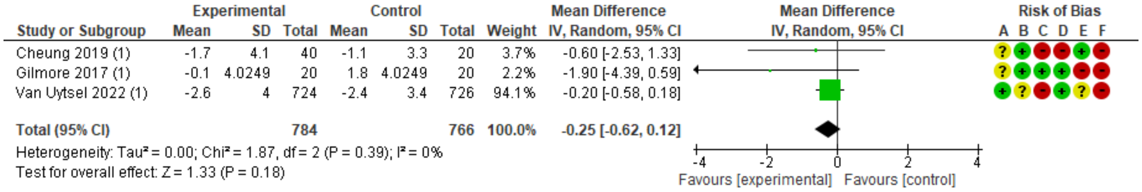

Risk of bias legend
(A) Randomization process
(B) Deviations from intended interventions
(C) Missing outcome data
(D) Measurement of the outcome
(E) Selection of the reported results
(F) Overall

Red with a - symbol : high risk of bias, yellow with a ? symbol : unclear risk of bias, and green with a + symbol : low risk of bias
