# Supplementary material for: Effectiveness of the Internet of Things for Improving Pregnancy and Postpartum Women’s Health in High-Income Countries: A Systematic Review and Meta-Analysis of Randomized Controlled Trials
Source: Healthcare (Basel). 2025 Aug 23;13(17):2103. doi: 10.3390/healthcare13172103 (PMC12428080; doi:10.3390/healthcare13172103)
Supplement: Supplementary file 1 [file healthcare-13-02103-s001.zip › Table S2. Search strategy.pdf]

Table S2. Search strategy.

Database: CENTRAL

Advanced Search/Search Manager
Date Run: 13/02/2023 21:19:25
#1 [mh "Mobile Applications"] OR [mh Internet] OR [mh "Cell Phone"] OR [mh "Computers, Handheld"] OR [mh "Medical Informatics Applications"] OR [mh "Therapy, Computer-Assisted"] 20744
#2 (App OR Apps):ti,ab 8365
#3 (Online OR Web OR Internet OR Digital*):ti 15528
#4 ((Online OR Web OR Internet OR Digital*) NEAR/3 (Based OR Application* OR Intervention* OR Program* OR Therap*)):ab 17993
#5 (Phone* OR Telephone* OR Smartphone* OR Cellphone* OR Smartwatch*):ti 6532
#6 ((Phone* OR Telephone* OR Smartphone* OR Cellphone* OR Smartwatch*) NEAR/3 (Based OR Application* OR Intervention* OR Program* OR Therap*)):ab 8506
#7 (Mobile Health OR mHealth OR m-Health OR eHealth OR e-Health OR eMental OR e-Mental):ti 2419
#8 ((Mobile Health OR mHealth OR M-Health OR eHealth OR e-Health OR eMental OR e-Mental) NEAR/3 (Based OR Application* OR Intervention* OR Program* OR Therap*)):ab 26186
#9 (Mobile* NEAR/3 (Based OR Application* OR Intervention* OR Device* OR Technolog*)):ti,ab 5783
#10 #1 OR #2 OR #3 OR #4 OR #5 OR #6 OR #7 OR #8 OR #9 75325
#11 [mh "Biosensing Techniques"] OR [mh "Wearable Electronic Devices"] OR [mh "Internet of Things"] OR [mh "Self-Help Devices"] OR [mh Smartphone] OR [mh Telemedicine] OR ("Internet of Things" OR IoT OR IoTs OR "Industrial Internet" OR Digital Device* OR Telemedicine* OR Tele-Medicine* OR Smart Device* OR "Web of Things" OR Smart Phone* OR Mobile Phone* OR Smart Ring* OR Smartring* OR Smart Wristband* OR Smart Watch* OR Smartwatch* OR Apple Watch* OR Wearable* OR Smart Home* OR SmartHome* OR "Digital Life" OR Smart Glass* OR Smartglass* OR Google Glass* OR Head Mounted Display* OR Head Worn Display* OR Head Up Display* OR Fitness Tracker* OR Activity Tracker*):ti,ab 17323
#12 #10 OR #11 83803
#13 [mh "Women's Health"] OR [mh "Women's Health Services"] OR [mh "Diabetes, Gestational"] OR [mh "Gestational Weight Gain"] OR [mh Women] OR [mh "Women, Working"] OR [mh "Hot Flashes"] OR [mh Infertility] OR [mh "Infertility, Female"] OR [mh Menstruation] OR [mh Menopause] OR [mh "Obesity, Maternal"] OR [mh Pregnancy] OR [mh "Pregnant Women"] OR (Pregnan* OR Women's Health* OR Working Women* OR Hot Flash* OR Hot Flush* OR Infertil* OR Menstrua* OR Menopaus* OR Maternal Obes* OR Gestational Diabet* OR Gestational Weight Gain*):ti,ab 140021
#14 #12 AND #13 in Trials 9197

Database: CINAHL

Advanced Search

S7 S5 AND S6 Limiters - Exclude MEDLINE records 657

S6 (MH ("Randomized Controlled Trials" OR "Double-Blind Studies" OR "Single-Blind Studies" OR "Random Assignment" OR "Pretest-Posttest Design" OR "Cluster Sample" OR "Placebos" OR "Crossover Design" OR "Comparative Studies") OR TI (Randomised OR Randomized OR Trial) OR AB (Random* OR (Control W5 Group) OR (Cluster W3 RCT)) OR (MH ("Sample Size") AND AB (Assigned OR Allocated OR Control)) OR PT (Randomized Controlled Trial)) NOT ((MH Animals+ OR MH (Animal Studies) OR TI (Animal Model*)) NOT MH (Human)) 955,445

S5 S3 AND S4 6,578

S4 S1 OR S2 219,937

S3 ((MH "Women's Health") OR (MH "Women's Health Services") OR (MH "Diabetes Mellitus, Gestational") OR (MH "Gestational Weight Gain") OR (MH "Women") OR (MH "Women, Working") OR (MH "Hot Flashes") OR (MH "Infertility") OR (MH "Menstruation") OR (MH "Menopause") OR (MH "Obesity, Maternal") OR (MH "Pregnancy") OR (MH "Expectant Mothers")) OR TI (Pregnan* OR Women's Health* OR Working Women* OR Hot Flash* OR Hot Flush* OR Infertil* OR Menstrua* OR Menopaus* OR Maternal Obes* OR Gestational Diabet* OR Gestational Weight Gain*) OR AB (Pregnan* OR Women's Health* OR Working Women* OR Hot Flash* OR Hot Flush* OR Infertil* OR Menstrua* OR Menopaus* OR Maternal Obes* OR Gestational Diabet* OR Gestational Weight Gain*) 419,488

S2 ((MH "Biosensing Techniques") OR (MH "Wearable Sensors+") OR (MH "Smart Glasses") OR (MH "Internet of Things") OR (MH "Assistive Technology Devices") OR (MH "Smartphone") OR (MH "Telehealth") OR (MH "Telemedicine")) OR TI ("Internet of Things" OR IoT OR IoTs OR "Industrial Internet" OR Digital Device* OR Telemedicine* OR Tele-Medicine* OR Smart Device* OR "Web of Things" OR Smart Phone* OR Mobile Phone* OR Smart Ring* OR Smartring* OR Smart Wristband* OR Smart Watch* OR Smartwatch* OR Apple Watch* OR Wearable* OR Smart Home* OR SmartHome* OR "Digital Life" OR Smart Glass* OR Smartglass* OR Google Glass* OR Head Mounted Display* OR Head Worn Display* OR Head Up Display* OR Fitness Tracker* OR Activity Tracker*) OR AB ("Internet of Things" OR IoT OR IoTs OR "Industrial Internet" OR Digital Device* OR Telemedicine* OR Tele-Medicine* OR Smart Device* OR "Web of Things" OR Smart Phone* OR Mobile Phone* OR Smart Ring* OR Smartring* OR Smart Wristband* OR Smart Watch* OR Smartwatch* OR Apple Watch* OR Wearable* OR Smart Home* OR SmartHome* OR "Digital Life" OR Smart Glass* OR Smartglass* OR Google Glass* OR Head Mounted Display* OR Head Worn Display* OR Head Up Display* OR Fitness Tracker* OR Activity Tracker*) 59,277

S1 ((MH "Mobile Applications") OR (MH "Internet") OR (MH "Internet-Based Intervention") OR (MH "Cellular Phone") OR (MH "Computers, Hand-Held") OR (MH "Therapy, Computer Assisted")) OR TI (App OR Apps) OR AB (App OR Apps) OR TI (Online OR Web OR Internet OR Digital*) OR AB ((Online OR Web OR Internet OR Digital*) N3 (Based OR Application* OR Intervention* OR Program* OR Therap*)) OR TI (Phone* OR Telephone* OR Smartphone* OR Cellphone* OR Smartwatch*) OR AB ((Phone* OR Telephone* OR Smartphone* OR Cellphone* OR Smartwatch*) N3 (Based OR Application* OR Intervention* OR Program* OR Therap*)) OR TI (Mobile Health OR mHealth OR m-Health OR eHealth OR e-Health OR eMental OR e-Mental) OR AB ((Mobile Health OR mHealth OR M-Health OR eHealth OR e-Health OR eMental OR e-Mental) N3 (Based OR Application* OR Intervention*

OR Program* OR Therap*)) OR TI (Mobile* N3 (Based OR Application* OR Intervention* OR Device* OR Technolog*)) OR AB (Mobile* N3 (Based OR Application* OR Intervention* OR Device* OR Technolog*)) 179,160

Database: ClinicalTrials.gov

Advanced Search
Condition or disease: Pregnant OR Pregnancy OR Women OR Hot Flash OR Infertility OR Menstruation OR Menopause OR Maternal OR Gestational
Other terms: Randomized OR RCT
Intervention/treatment: App OR Online OR Web OR Internet OR Digital OR Mobile OR Phone Application OR Smartphone OR Cellphone OR Smartwatch OR mHealth OR eHealth OR "Internet of Things" OR Telemedicine OR Wearable OR Head Mounted Display OR Head Up Display OR Tracker
1086 Studies found

Database: Embase

Database: Embase <1980 to 2023 Week 06>

1 exp Mobile Application/ or Internet/ or exp Mobile Phone/ or Text Messaging/ or Personal Digital Assistant/ or Computer Assisted Therapy/ or (App or Apps).ti,ab. or (Online or Web or Internet or Digital*).ti. or ((Online or Web or Internet or Digital*) adj3 (Based or Application* or Intervention* or Program* or Therap*)).ab. or (Phone* or Telephone* or Smartphone* or Cellphone* or Smartwatch*).ti. or ((Phone* or Telephone* or Smartphone* or Cellphone* or Smartwatch*) adj3 (Based or Application* or Intervention* or Program* or Therap*)).ab. or (Mobile Health or mHealth or m-Health or eHealth or e-Health or eMental or e-Mental).ti. or ((Mobile Health or mHealth or m-Health or eHealth or e-Health or eMental or e-Mental) adj3 (Based or Application* or Intervention* or Program* or Therap*)).ab. or (Mobile* adj3 (Based or Application* or Intervention* or Device* or Technolog*)).ti,ab. (416388)

2 Online Application/ or Smartphone Application/ or exp Wearable Electronic Devices/ or "Internet of Things"/ or Self Help Device/ or Smartphone/ or Telemedicine/ or ("Internet of Things" or IoT or IoTs or "Industrial Internet" or Digital Device* or Telemedicine* or Tele-Medicine* or Smart Device* or "Web of Things" or Smart Phone* or Mobile Phone* or Smart Ring* or Smartring* or Smart Wristband* or Smart Watch* or Smartwatch* or Apple Watch* or Wearable* or Smart Home* or SmartHome* or "Digital Life" or Smart Glass* or Smartglass* or Google Glass* or Head Mounted Display* or Head Worn Display* or Head Up Display* or Fitness Tracker* or Activity Tracker*).ti,ab. (122768)

3 exp Women's Health/ or Female Worker/ or exp Pregnancy Diabetes Mellitus/ or Gestational Weight Gain/ or Hot Flush/ or Infertility/ or Female Infertility/ or Menstruation/ or exp Menopause/ or Maternal Obesity/ or Pregnancy/ or Pregnant Woman/ or (Pregnan* or Women's Health* or Working Women* or Hot Flash* or Infertil* or Menstrua* or Menopaus* or Maternal Obes* or Gestational Diabet* or Gestational Weight Gain*).ti,ab. (1225851)

4 Randomized controlled trial/ or Controlled clinical study/ or randomization/ or intermethod comparison/ or double blind procedure/ or human experiment/ or (random$ or placebo or (open adj label) or ((double or single or doubly or singly) adj (blind or blinded or blindly)) or parallel group$1 or crossover or cross over or ((assign$ or match or matched or allocation) adj5 (alternate or group$1 or intervention$1 or patient$1 or subject$1 or participant$1)) or assigned or allocated or (controlled adj7 (study or design or trial)) or volunteer or volunteers).ti,ab. or (compare or compared or comparison or trial).ti. or ((evaluated or evaluate or evaluating or assessed or assess) and (compare or compared or comparing or comparison)).ab. (6042208)

5 (random$ adj sampl$ adj7 ("cross section$" or questionnaire$1 or survey$ or database$1)).ti,ab. not (comparative study/ or controlled study/ or randomi?ed controlled.ti,ab. or randomly assigned.ti,ab.) (9157)

6 Cross-sectional study/ not (randomized controlled trial/ or controlled clinical study/ or controlled study/ or (randomi?ed controlled or control group$1).ti,ab.) (329671)

7 (((case adj control$) and random$) not randomi?ed controlled).ti,ab. (20870)

8 (Systematic review not (trial or study)).ti. (240859)

9 (nonrandom$ not random$).ti,ab. (18251)

10 ("Random field$" or (random cluster adj3 sampl$)).ti,ab. (4333)

11 (review.ab. and review.pt.) not trial.ti. (1061010)

12 "we searched".ab. and (review.ti. or review.pt.) (46695)

13 ("update review" or (databases adj4 searched)).ab. (58001)

14 (rat or rats or mouse or mice or swine or porcine or murine or sheep or lambs or pigs or

piglets or rabbit or rabbits or cat or cats or dog or dogs or cattle or bovine or monkey or monkeys or trout or marmoset$1).ti. and animal experiment/ (1150244)

15 Animal experiment/ not (human experiment/ or human/) (2387530)

16 or/5-15 (4050979)

17 4 not 16 (5336424)

18 (1 or 2) and 3 and 17 (3682)

19 limit 18 to (conference abstracts or embase) (3021)

Database: MEDLINE

Ovid MEDLINE(R) ALL <1946 to February 10, 2023>
1 Mobile Applications/ or exp Internet/ or exp Cell Phone/ or exp Computers, Handheld/ or Medical Informatics Applications/ or Therapy, Computer-Assisted/ or (App or Apps).ti,ab. or (Online or Web or Internet or Digital*).ti. or ((Online or Web or Internet or Digital*) adj3 (Based or Application* or Intervention* or Program* or Therap*)).ab. or (Phone* or Telephone* or Smartphone* or Cellphone* or Smartwatch*).ti. or ((Phone* or Telephone* or Smartphone* or Cellphone* or Smartwatch*) adj3 (Based or Application* or Intervention* or Program* or Therap*)).ab. or (Mobile Health or mHealth or m-Health or eHealth or e-Health or eMental or e-Mental).ti. or ((Mobile Health or mHealth or M-Health or eHealth or e-Health or eMental or e-Mental) adj3 (Based or Application* or Intervention* or Program* or Therap*)).ab. or (Mobile* adj3 (Based or Application* or Intervention* or Device* or Technolog*)).ti,ab. (328538)
2 Biosensing Techniques/ or exp Wearable Electronic Devices/ or Internet of Things/ or Self-Help Devices/ or Smartphone/ or Telemedicine/ or ("Internet of Things" or IoT or IoTs or "Industrial Internet" or Digital Device* or Telemedicine* or Tele-Medicine* or Smart Device* or "Web of Things" or Smart Phone* or Mobile Phone* or Smart Ring* or Smartring* or Smart Wristband* or Smart Watch* or Smartwatch* or Apple Watch* or Wearable* or Smart Home* or SmartHome* or "Digital Life" or Smart Glass* or Smartglass* or Google Glass* or Head Mounted Display* or Head Worn Display* or Head Up Display* or Fitness Tracker* or Activity Tracker*).ti,ab. (163553)
3 exp Women's Health/ or Women's Health Services/ or Diabetes, Gestational/ or Gestational Weight Gain/ or Women/ or Women, Working/ or Hot Flashes/ or Infertility/ or Infertility, Female/ or Menstruation/ or exp Menopause/ or Obesity, Maternal/ or Pregnancy/ or Pregnant Women/ or (Pregnan* or Women's Health* or Working Women* or Hot Flash* or Hot Flush* or Infertil* or Menstrua* or Menopaus* or Maternal Obes* or Gestational Diabet* or Gestational Weight Gain*).ti,ab. (1330912)
4 ((Randomized Controlled Trial or Controlled Clinical Trial).pt. or (Randomi?ed or Placebo or Randomly or Trial or Groups).ab. or Drug Therapy.fs.) not (exp Animals/ not Humans.sh.) (4888013)
5 (1 or 2) and 3 and 4 (2821)

Database: PsycINFO

Database: APA PsycInfo <1806 to January Week 5 2023>

1 Mobile Applications/ or exp Internet/ or exp Mobile Phones/ or Computer Assisted Therapy/ or Online Therapy/ or Digital Interventions/ or Mobile Health/ or (App or Apps).ti,ab. or (Online or Web or Internet or Digital*).ti. or ((Online or Web or Internet or Digital*) adj3 (Based or Application* or Intervention* or Program* or Therap*)).ab. or (Phone* or Telephone* or Smartphone* or Cellphone* or Smartwatch*).ti. or ((Phone* or Telephone* or Smartphone* or Cellphone* or Smartwatch*) adj3 (Based or Application* or Intervention* or Program* or Therap*)).ab. or (Mobile Health or mHealth or m-Health or eHealth or e-Health or eMental or e-Mental).ti. or ((Mobile Health or mHealth or M-Health or eHealth or e-Health or eMental or e-Mental) adj3 (Based or Application* or Intervention* or Program* or Therap*)).ab. or (Mobile* adj3 (Based or Application* or Intervention* or Device* or Technolog*)).ti,ab. (116589)

2 Wearable Devices/ or Smartphones/ or Telemedicine/ or Mobile Devices/ or Tablet Computers/ or ("Internet of Things" or IoT or IoTs or "Industrial Internet" or Digital Device* or Telemedicine* or Tele-Medicine* or Smart Device* or "Web of Things" or Smart Phone* or Mobile Phone* or Smart Ring* or Smartring* or Smart Wristband* or Smart Watch* or Smartwatch* or Apple Watch* or Wearable* or Smart Home* or SmartHome* or "Digital Life" or Smart Glass* or Smartglass* or Google Glass* or Head Mounted Display* or Head Worn Display* or Head Up Display* or Fitness Tracker* or Activity Tracker*).ti,ab. (22193)

3 Human Females/ or Working Women/ or Gestational Diabetes/ or Infertility/ or Menstruation/ or Menopause/ or Pregnancy/ or (Pregnan* or Women's Health* or Working Women* or Hot Flash* or Hot Flush* or Infertil* or Menstrua* or Menopaus* or Maternal Obes* or Gestational Diabet* or Gestational Weight Gain*).ti,ab. (162496)

4 exp Clinical Trials/ or Placebo/ or (random* or sham or placebo* or ((singl* or doubl*) adj (blind* or dumm* or mask*)) or ((tripl* or trebl*) adj (blind* or dumm* or mask*)) or (control* adj3 (study or studies or trial* or group*)) or Nonrandom* or non random* or non-random* or quasi-random* or quasirandom* or allocated or ((open label or open-label) adj5 (study or studies or trial*)) or ((equivalence or superiority or non-inferiority or noninferiority) adj3 (study or studies or trial*)) or ((pragmatic or practical) adj3 trial*) or ((quasiexperimental or quasi-experimental) adj3 (study or studies or trial*)) or (phase adj3 (III or "3") adj3 (study or studies or trial*))).ti,ab,hw. (395145)

5 (1 or 2) and 3 and 4 (504)

Database: PubMed

(App[TIAB] OR Apps[TIAB] OR Online[TI] OR Web[TI] OR Internet[TI] OR Digital*[TI] OR ((Online[TIAB] OR Web[TIAB] OR Internet[TIAB] OR Digital*[TIAB]) AND (Based[TIAB] OR Application*[TIAB] OR Intervention*[TIAB] OR Program*[TIAB] OR Therap*[TIAB])) OR Phone*[TI] OR Telephone*[TI] OR Smartphone*[TI] OR Cellphone*[TI] OR Smartwatch*[TI] OR ((Phone*[TIAB] OR Telephone*[TIAB] OR Smartphone*[TIAB] OR Cellphone*[TIAB] OR Smartwatch*[TIAB]) AND (Based[TIAB] OR Application*[TIAB] OR Intervention*[TIAB] OR Program*[TIAB] OR Therap*[TIAB])) OR Mobile Health[TI] OR mHealth[TI] OR m-Health[TI] OR eHealth[TI] OR e-Health[TI] OR eMental[TI] OR e-Mental[TI] OR ((Mobile Health[TIAB] OR mHealth[TIAB] OR M-Health[TIAB] OR eHealth[TIAB] OR e-Health[TIAB] OR eMental[TIAB] OR e-Mental[TIAB]) AND (Based[TIAB] OR Application*[TIAB] OR Intervention*[TIAB] OR Program*[TIAB] OR Therap*[TIAB])) OR (Mobile*[TIAB] AND (Based[TIAB] OR Application*[TIAB] OR Intervention*[TIAB] OR Device*[TIAB] OR Technolog*[TIAB])) OR "Internet of Things"[TIAB] OR IoT[TIAB] OR IoTs[TIAB] OR "Industrial Internet"[TIAB] OR Digital Device*[TIAB] OR Telemedicine*[TIAB] OR Tele-Medicine*[TIAB] OR Smart Device*[TIAB] OR "Web of Things"[TIAB] OR Smart Phone*[TIAB] OR Mobile Phone*[TIAB] OR Smart Ring*[TIAB] OR Smartring*[TIAB] OR Smart Wristband*[TIAB] OR Smart Watch*[TIAB] OR Smartwatch*[TIAB] OR Apple Watch*[TIAB] OR Wearable*[TIAB] OR Smart Home*[TIAB] OR SmartHome*[TIAB] OR "Digital Life"[TIAB] OR Smart Glass*[TIAB] OR Smartglass*[TIAB] OR Google Glass*[TIAB] OR Head Mounted Display*[TIAB] OR Head Worn Display*[TIAB] OR Head Up Display*[TIAB] OR Fitness Tracker*[TIAB] OR Activity Tracker*[TIAB]) AND (Pregnan*[TIAB] OR Women's Health*[TIAB] OR Working Women*[TIAB] OR Hot Flash*[TIAB] OR Hot Flush*[TIAB] OR Infertil*[TIAB] OR Menstrua*[TIAB] OR Menopaus*[TIAB] OR Maternal Obes*[TIAB] OR Gestational Diabet*[TIAB] OR Gestational Weight Gain*[TIAB]) AND (Randomized[TIAB] OR Randomised[TIAB] OR Placebo[TIAB] OR Randomly[TIAB] OR Trial[TIAB] OR Groups[TIAB]) NOT MEDLINE[SB]
714

Database: WHO ICTRP

Advanced Search
Pregnant OR Pregnancy OR Women OR Hot Flash OR Infertility OR Menstruation OR Menopause OR Maternal OR Gestational ***in the Condition***
App OR Online OR Web OR Internet OR Digital OR Mobile OR Phone Application OR Smartphone OR Cellphone OR Smartwatch OR mHealth OR eHealth OR "Internet of Things" OR Telemedicine OR Wearable OR Head Mounted Display OR Head Up Display OR Tracker ***in the Intervention***
Recruitment status is ALL
435 records for 433 trials found
