# Supplementary material for: Effectiveness of the Internet of Things for Improving Pregnancy and Postpartum Women’s Health in High-Income Countries: A Systematic Review and Meta-Analysis of Randomized Controlled Trials
Source: Healthcare (Basel). 2025 Aug 23;13(17):2103. doi: 10.3390/healthcare13172103 (PMC12428080; doi:10.3390/healthcare13172103)
Supplement: Supplementary file 1 [file healthcare-13-02103-s001.zip › Table S3. Summary of findings.pdf]

Table S3. Summary of findings.

Outcomes	Anticipated absolute effects* (95% CI)		Relative effect (95% CI)	№ of participants (studies)	Certainty of the evidence (GRADE)	Comments
	**Risk with placebo**	**Risk with IoT**				
Composite pregnancy morbidity	333 per 1,000	**283 per 1,000** (173 to 457)	**RR 0.85** (0.52 to 1.37)	150 (1 RCT)	⨁◯◯◯ Very low[b,c]	
Miscarriage ≤22 weeks	0 per 1,000	**0 per 1,000** (0 to 0)	**RR 4.62** (0.23 to 94.64)	150 (1 RCT)	⨁◯◯◯ Very low[b,c]	
Gestational diabetes	167 per 1,000	**128 per 1,000** (58 to 278)	**RR 0.77** (0.35 to 1.67)	150 (1 RCT)	⨁◯◯◯ Very low[b,c]	
Diabetes at 4-12 weeks postpartum	200 per 1,000	**182 per 1,000** (32 to 1,000)	**RR 0.91** (0.16 to 5.30)	21 (1 RCT)	⨁◯◯◯ Very low[a,b,c]	
Preeclampsia or gestational hypertension	125 per 1,000	**78 per 1,000** (29 to 205)	**RR 0.62** (0.23 to 1.64)	150 (1 RCT)	⨁◯◯◯ Very low[b,c]	
Preterm labor ≤37 weeks	69 per 1,000	**90 per 1,000** (30 to 270)	**RR 1.29** (0.43 to 3.89)	150 (1 RCT)	⨁◯◯◯ Very low[b,c]	
Birthweight ≤2500 g	28 per 1,000	**64 per 1,000** (13 to 320)	**RR 2.31** (0.46 to 11.52)	150 (1 RCT)	⨁◯◯◯ Very low[b,c]	

Outcomes	Anticipated absolute effects* (95% CI)		Relative effect (95% CI)	№ of participants (studies)	Certainty of the evidence (GRADE)	Comments
	**Risk with placebo**	**Risk with IoT**				

***The risk in the intervention group** (and its 95% confidence interval) was based on the assumed risk in the comparison group and the **relative effect** of the intervention (and its 95% CI).

CI: confidence interval; **MD:** mean difference; **RR:** risk ratio

GRADE Working Group grades of evidence: High certainty: we are very confident that the true effect lies close to that of the estimate of the effect. **Moderate certainty:** we are moderately confident in the effect estimate: the true effect is likely to be close to the estimate of the effect, but there is a possibility that it is substantially different. **Low certainty:** our confidence in the effect estimate is limited: the true effect may be substantially different from the estimate of the effect. **Very low certainty:** we have very little confidence in the effect estimate: the true effect is likely to be substantially different from the estimate of effect.

a. unclear or high risk of bias for the randomization process, b. estimate based on a wide confidence interval crossing the line of no effect, c. small sample size
