# Supplementary material for: Effectiveness of the Internet of Things for Improving Pregnancy and Postpartum Women’s Health in High-Income Countries: A Systematic Review and Meta-Analysis of Randomized Controlled Trials
Source: Healthcare (Basel). 2025 Aug 23;13(17):2103. doi: 10.3390/healthcare13172103 (PMC12428080; doi:10.3390/healthcare13172103)
Supplement: Supplementary file 1 [file healthcare-13-02103-s001.zip › Table S4. Additional characteristics of the included studies.pdf]

Table S4. Additional characteristics of the included studies.

Author, Year, Country [Study design]	N (intervention, Comparison) [Attrition Rate (intervention, Comparison)]	Inclusion criteria / Exclusion criteria	Intervention / Comparison	Outcomes reported
Gilmore et al., 2017, United States of America [Individual randomized controlled trial (pilot)]	40 (20 vs 20) [12.5% (5%, 20%)]	**Inclusion criteria:** Postpartum women aged >18 years who delivered within the past 8 weeks, had a BMI ranging from 25–40 kg/m^2, were eligible for WIC postpartum services, and could speak English.	**Intervention:** The standard care provided by the WIC program, supplemented with personalized weight management utilizing a smartphone application termed E-Moms. Participants underwent a personalized lifestyle intervention facilitated by the SmartLoss application. To monitor both weight fluctuations and physical activity levels, participants were furnished with a Body Trace scale and a Fitbit Zip accelerometer.	Primary: change in weight (kg), Secondary: body fat percentage, waist circumference (cm), hip circumference (**cm**), and waist-to-hip ratio
		**Exclusion criteria:** Participants enrolled in the Nurse Family Partnership program, with a history of multiple gestation during the index pregnancy, psychiatric	**Comparison:** The standard care (WIC Moms), constituting standardized advice and services for postpartum nutrition and weight management through their WIC clinic. Participants in this group did not	

		disorders, chronic diseases affecting body weight, appetite, or intake, type 1 diabetes mellitus, or utilizing medications or supplements to facilitate weight loss.	receive a prescribed dietary regimen or personalized weight management services from the research team.	
Cheung et al., 2019, Australia [Individual randomized controlled trial (pilot)]	60 (40, 20)<br><br>[55% (52.5%, 60%)]	**Inclusion criteria:**<br>Women with GDM diagnosed according to the 1998 Australasian Diabetes in Pregnancy Society Criteria, with a fasting glucose level of ≥5.5 mmol/L and/or a 2 h glucose level of ≥8 mmol/L after a 75 g oral glucose tolerance test, aged ≥18 years, ownership of a smartphone with text messaging capability, internet access, proficiency in English adequate for reading text messages, and physical capability for moderate-intensity physical activity.	**Intervention:**<br>A text messaging intervention (TEXT ME) program linked to a Fitbit Flex® active monitor that enabled tracking of activity and further customization of text messages that were sent during two 30-min lifestyle counselling sessions.	(i) Attendance for the post-partum GTT within 12 weeks post-partum;<br>(ii) Adherence to physical activity recommendations, constituting 30 min of moderate intensity physical activity at least 5 days a week as a self-reported outcome, along with achieving a daily step count of 10,000 recorded by pedometer count, assessed at the 6-month mark;<br>(iii) Achievement of dietary macronutrient recommendations regarding fat and fiber intake,

		**Exclusion criteria:**<br>Not mentioned.	**Comparison:**<br>Only a booklet "Life after Gestational Diabetes" developed by Diabetes Australia.	including a dietary fat intake of ≤30% of total daily caloric intake, saturated fat consumption below 10%, and the consumption of 15 g of fiber per 1,000 calories, evaluated at the 6-month interval and;<br>(iv) Evaluation of the change in self-reported weight (kg) recorded at the 6-month follow-up.
Sung et al, 2019, South Korea [Individual randomized controlled trial (pilot)]	21 (11, 10)<br><br>[9.5% (10%, 9.1%)]	**Inclusion criteria:**<br>Singleton pregnant women diagnosed with GDM at 24–28 weeks of gestation.	**Intervention:**<br>Mobile management (MM) group: standard antenatal care and tailored mobile health care services by the mobile phone application designed by the study. Participants in the MM group were given monitoring system devices, including a glucometer with Bluetooth connectivity and an accelerometer to detect physical activity levels.	Obstetrical outcomes: GA at delivery, birth weight (kg), small for GA, large for GA, cesarean section. Metabolic outcomes: Maternal BMI, weight (kg), body fat (%), HOMA-IR.

		**Exclusion criteria:**<br>Patients after 30 weeks of gestation, those with pre-gestational diabetes, those who do not understand Korean, those who were unfamiliar with mobile phone, those who do not have access to a mobile phone, and those who were already receiving services from another mobile health service.	**Comparison:**<br>Conventional management (CM) group: standard antenatal care from obstetricians and endocrinologists.	
Chen et al., 2023, Taiwan [Individual randomized controlled trial]	92 (46, 46)<br><br>[13.0% (19.5%, 6.5%)]	**Inclusion criteria:**<br>Pregnant women with a BMI ≥25 kg/m^2 during the antenatal care services, within less than 17 weeks of gestation, aged ≥20 years, proficient in Mandarin Chinese, and possessing basic technological literacy, such as using smartphones and application interfaces.	**Intervention:**<br>The intervention group received the MyHealthyWeight (MHW) application, compatible with both Android and iOS platforms, along with a wrist-worn Mi Band 5 (WAT).<br>The Mi Band 5, functioning as a wearable activity tracker, prompted participants to wear the device for a minimum of 16 h daily. Participants were prescribed a physical activity goal of 8,500 steps per day.	Primary: rate of excessive weekly GWG (kg/week), rate of excessive total GWG (kg), changes and trajectories of GWG (kg) in both groups throughout pregnancy<br>Secondary: factors related to GWG (kg) among overweight and obese pregnant women and changes in daily steps during pregnancy.

		**Exclusion criteria:**<br>Pregnant women with a diagnosis of eating-related disorders, diabetes, and/or medical conditions that influence body weight.	**Comparison:**<br>The control group received standard antenatal care consisting of routine monitoring of maternal and fetal health.	
Gonzalez-Plaza et al., 2022, Spain [Individual randomized controlled trial]	150 (78, 72)<br><br>[23.3% (19.2%, 27.7%)]	**Inclusion criteria:**<br>Pregnant women exhibiting pre-pregnancy obesity (defined as having a BMI ≥30 kg/m^2 according to the World Health Organization classification) during the 12–18 weeks of pregnancy, who attended hospital obstetric clinics during prenatal care, maintaining a singleton pregnancy, aged ≥18 years, possessing an Android smartphone or iPhone (iOS) with an internet connection.	**Intervention:**<br>The intervention group was provided with a smartband (Mi Band 2). Pregnant women were advised to engage in a daily regimen of 10,000 steps, corresponding to at least 30 min of moderate physical activity per day, as per the guidelines set forth by the American College of Obstetricians and Gynecologists. The smartband was synchronized with the freely accessible Mi Fit application, compatible with Android and iOS platforms. The women confirmed objective fulfillment by alerts and notifications from the Mi Fit application and the smartband.<br>The application for receiving health counseling and support from a midwife (Hangouts (Google LLC) was also provided.	(i) Primary: GWG (Gestation Weight Gain (kg)) and total physical activity.<br>(ii) Secondary: the incidence of maternal complications, Incidence of birth induction, type of delivery, and unplanned cesarean section.<br>Incidence of birth induction, type of delivery, and unplanned cesarean section.<br>Incidence of perinatal complications

		**Exclusion criteria:**<br>Pregnant women who were already using a physical activity and weight monitoring application. Pregnant women who have been diagnosed with psychiatric disorders, endocrine–metabolic disorders, or chronic hypertension; pregnant women with a contraindication for performing exercise or mobility problems that do not allow moderate walking; and women with language difficulties in understanding Spanish	**Comparison:**<br>The control group received oral information and written support material.<br>Pregnant women were advised to engage in 30 min of moderate physical activity per day over the week (≥5 days), a GWG between 5–9 kg, and a balanced (Mediterranean) diet of 1,800 kcal.	
Lim et al., 2021, Singapore [Individual randomized controlled trial]	200 (101, 99)<br><br>[5.5% (4.9%, 6.1%)]	**Inclusion criteria:**<br>Postpartum women aged ≥21 years, diagnosed with GDM between 24–34 weeks of pregnancy using a 75-g, three time-point OGTT according to the 2013 World Health Organization criteria, were included. Women needed to	**Intervention:**<br>Women assigned to the intervention group were directed to download the Nutritionist Buddy (nBuddy) application immediately postpartum before discharge and were trained in its use by a study team member.<br>Participants used the application to pursue personalized weight targets. Participants were	Primary: the percentage of women who regained their first trimester weight by four months postpartum if their first trimester BMI was ≤23 kg/m², or achieved a weight loss of at least 5% from their first trimester weight if their

	independently possess and operate a smartphone. Additionally, their weight in the first trimester had to be recorded by or before 13 weeks of gestation.	instructed to log their daily food intake using the application's database of over 11,000 local foods.<br>The step-counting feature enabled users to monitor their physical activity. The study produced 16 short video clips, each lasting 3 min, covering topics, including diet, exercise, emotional health for new mothers, and the benefits of breastfeeding and weaning for babies.<br>The functions were available.	first trimester BMI was >23 kg/m².<br><br>Secondary:<br>(i) A 75-g, 2-h OGTT, HbA1c, C-peptide, homeostatis model assessment of insulin resistance, lipid profiles, liver function, high-sensitivity C-reactive protein, and interleukin-6.<br>(ii) Mean absolute weight loss (kg).<br>(iii) Breastfeeding status.<br>(iv) Blood pressure.<br>(v) Right hand grip strength (kg) and waist circumference (cm).<br>(vi) heiQ, self-efficacy, and RAND-12 questionnaire.<br>(vii) Caloric and macronutrient intake assessed by a 3-day food diary.
	**Exclusion criteria:**<br>Women with pre-existing type 1 or type 2 diabetes mellitus, and those who gave birth before 36 weeks.	**Comparison:**<br>Women in the standard care group had a 6-week postpartum appointment with a clinician for a routine checkup, including dietary advice and a repeat OGTT.	

Van Uytsel et al., 2022, Belgium [Individual randomized controlled trial]	1075 (551, 524) [23.7% (22.0%, 25.6%)]	**Inclusion criteria:** Women who are at least 18 years of age and have excess GWG according to the 2009 National Academy of Medicine guidelines.	**Intervention:** Four face-to-face lifestyle coaching sessions using a smartphone application to support the participants in behavior change. A Bluetooth connection was set up with an activity tracker (Withings Go) and a weighing scale (Withings Body+).	Weight retention (kg), fat percentage, waist and hip circumference (cm), energy intake, improved physical activity (an increase of 700 MET-minutes/week), and improved sedentary time (a decrease of 1 sedentary hours/day).
		**Exclusion criteria:** Women were excluded if they needed complex medical diets, had a history of or were planning bariatric surgery, had chronic conditions, including type 2 diabetes or significant psychiatric disorders, or experienced a stillbirth in their last pregnancy. Participants were excluded from the analysis if body weight and anthropometric data were missing at both 6 weeks and 6 months postpartum.	**Comparison:** Control group participants received standard antenatal and postnatal care.	
