# Supplementary material for: Effectiveness of the Internet of Things for Improving Pregnancy and Postpartum Women’s Health in High-Income Countries: A Systematic Review and Meta-Analysis of Randomized Controlled Trials
Source: Healthcare (Basel). 2025 Aug 23;13(17):2103. doi: 10.3390/healthcare13172103 (PMC12428080; doi:10.3390/healthcare13172103)
Supplement: Supplementary file 1 [file healthcare-13-02103-s001.zip › Table S5. Fat change_rev.pdf]

Table S5. Body fat change.

Author Name	IoT Device & Application	Physical Activity Tracked by IoT (Specified in the article)	Intervention Details	Method of Measuring Body Fat
Gilmore et al., 2017	Wearable activity monitor (Fitbit Zip®), BodyTrace smart scale & SmartLoss® mobile application	Step count	The intervention, called E-Moms, was a personalized mobile health program delivered through the SmartLoss® smartphone application. Participants in the E-Moms intervention group were provided with a BodyTrace® Smart Scale and a Fitbit Zip® Accelerometer to track their weight and step count.<br>The interventionist (a registered dietitian) monitored the weight and activity data in near real-time through the SmartLoss® web portal. If weight data deviated from the target weight loss zone for approximately three consecutive days, participants received personalized advice via phone calls, emails, or text messages.	The change in body fat percentage was calculated as the difference between 6-8 weeks postpartum (baseline measurement) and approximately 22-24 weeks postpartum.<br>Body fat percentage was measured in triplicate using a Tanita SC-240 foot-to-foot bioelectric impedance analysis (BIA).

Sung et al., 2019	Glucometer, Accelerometer& Mobile application	physical activity level	Mobile healthcare services in the Mobile Management (MM) group included a custom-designed mobile application (developed by Huraypositive Inc.) that allowed patients to record and transmit their blood glucose levels and dietary intake. Participants were provided with a Bluetooth-enabled glucometer and an accelerometer to monitor physical activity. A multidisciplinary healthcare team, including endocrinologists, nurses, and nutritionists, reviewed the transmitted data and provided tailored coaching via the application. Patients received regular messages (about 3.7 per week) regarding diet, exercise, and weight management	The change in body fat percentage was calculated as the difference between approximately 27 weeks of gestation (baseline measurement) and 4-12 weeks postpartum.<br>Body fat percentage was automatically measured using a bioelectrical impedance analysis (BIA).
Van Uytsel et al., 2022	Wearable Activity Monitor (Withings Go), weighing scale (Withings Body+) & Mobile application	NA	The INTER-ACT intervention consisted of a combination of face-to-face lifestyle coaching and an e-health-supported smartphone application. The face-to-face coaching sessions were conducted at 6 weeks, 8 weeks, 12 weeks, and 6 months postpartum, focusing on nutrition, physical activity, and mental well-being. In each session, motivational interviewing (Ask-Tell-Ask method, **transtheoretical model**), **behavior change techniques** (goal setting, action planning, self-monitoring, encouragement, etc.), and **shared decision-making** played a central role.<br>A Bluetooth connection was set up with an activity tracker (Withings Go) and a weighing scale (Withings Body+). The application facilitated self-monitoring, goal-setting and sent tailored motivational messages.	The change in body fat percentage was calculated as the difference between 6 weeks postpartum (baseline measurement) and 6 months postpartum (post-intervention measurement).<br>A Tanita MC-780 SMA bioelectric impedance analysis (BIA) device was used for the measurements.
