# Supplementary material for: Effectiveness of the Internet of Things for Improving Pregnancy and Postpartum Women’s Health in High-Income Countries: A Systematic Review and Meta-Analysis of Randomized Controlled Trials
Source: Healthcare (Basel). 2025 Aug 23;13(17):2103. doi: 10.3390/healthcare13172103 (PMC12428080; doi:10.3390/healthcare13172103)
Supplement: Supplementary file 1 [file healthcare-13-02103-s001.zip › Table S6. Gestational weight gain_rev.pdf]

Table S6. Gestational weight gain.

Author Name	IoT Device & Application	Physical Activity Tracked by IoT (Specified in the article)	Intervention Details	Method of Measuring Gestational Weight
Chen et al., 2023	Wearable activity tracker (Mi Band 5) & Mobile application (MyHealthy Weight)	Step count	The intervention in this study was designed based on the **Social Cognitive Theory (SCT)**.<br>Pregnant women were instructed to wear the Mi Band 5 for at least 16 hours per day and achieve the target step count of 8,500 steps per day. The Mi Band 5 collected data on the pregnant women's step count and physical activity levels, while the MyHealthyWeight application analyzed the data and provided feedback.<br>The MyHealthyWeight application provided features for weight management, including weight, diet, and physical activity tracking, as well as goal setting, self-monitoring, reminders, and a reward system.	Gestational weight gain (kg) was calculated as the difference between the weight before pregnancy and the weight at 34–36 weeks gestation (third trimester).<br>Before pregnancy: weight from electronic health records.<br>At 34–36 weeks gestation: the weight data measured during routine hospital check-ups.

Gonzalez-Plaza et al., 2022	Wearable Activity Monitor (Mi Band 2) & Mobile application (Mi Fit)	Step count	The intervention in this study was designed based on the **Social Cognitive Theory (SCT)**.<br>The smartband was connected to the Mi Fit application, available for Android and iOS. The midwife guided participants in the intervention group to set step and weight goals using application notifications and alerts. The smartband vibrated during inactivity and rewarded goal achievements. Participants confirmed goal fulfillment through alerts and notifications from the Mi Fit application and the Mi Band 2.<br>The application used for receiving health counseling and support from the midwife was Hangouts (Google LLC). Pregnant women received personalized information (physiological changes in the mother and fetus, healthy eating habits, weight gain, and physical activity) through SMS text messages or videos sent twice a week by the midwife.	Gestational weight gain (kg) was calculated as the difference between the weight at the time of recruitment and the weight at 35–37 weeks of pregnancy.<br>At the time of recruitment: Self-reported.<br>At 35–37 weeks of pregnancy: Measured by a midwife during a consultation using a Seca 704 scale, with participants wearing clothes but without shoes.
