# Supplementary material for: Effectiveness of the Internet of Things for Improving Pregnancy and Postpartum Women’s Health in High-Income Countries: A Systematic Review and Meta-Analysis of Randomized Controlled Trials
Source: Healthcare (Basel). 2025 Aug 23;13(17):2103. doi: 10.3390/healthcare13172103 (PMC12428080; doi:10.3390/healthcare13172103)
Supplement: Supplementary file 1 [file healthcare-13-02103-s001.zip › Table S7. Weight change of postpartum women_rev.pdf]

**Table S7. Weight change of postpartum women.**

| Author Name | IoT Device & Application | Physical Activity Tracked by IoT (Specified in the article) | Intervention Details | Method of Measuring Weight |
|---|---|---|---|---|
| Gilmore et al., 2017 | Wearable activity monitor (Fitbit Zip®), BodyTrace smart scale & SmartLoss® mobile application | Step count | The intervention, called E-Moms, was a personalized mobile health program delivered through the SmartLoss® smartphone application. Participants in the E-Moms intervention group were provided with a BodyTrace® Smart Scale and a Fitbit Zip® Accelerometer to track their weight and step count.<br>The interventionist (a registered dietitian) monitored the weight and activity data in near real-time through the SmartLoss® web portal. If weight data deviated from the target weight loss zone for approximately three consecutive days, participants received personalized advice via phone calls, emails, or text messages. | Weight change (kg) was calculated as the difference between 6–8 weeks postpartum and 22–24 weeks postpartum.<br>Weight was measured using a Tanita BWB-800S scale while participants wore underwear or light clothing. |

| | | | | |
|---|---|---|---|---|
| Cheung et al., 2019 | Wearable activity monitor (Fitbit Flex®) & Mobile application | Step count | Participants received customized text messages tailored to their individual needs, focusing on physical activity, nutrition, and general health.These messages were adapted from the TEXT ME trial to better suit women after gestational diabetes (GDM). In particular, the content was customized to enhance information on physical activity, nutrition, and maternal and child health, encouraging participants to improve their lifestyle. Furthermore, these messages were personalized based on data collected from a wearable activity monitor (Fitbit Flex®), which tracked daily step counts. The step data was used to set adaptive step targets, with goals adjusting weekly according to previous activity levels, up to a maximum of 10,000 steps per day.<br>The intervention included diet counseling provided by a dietitian. | Weight change (kg) was calculated as the difference between 10–12 weeks postpartum (baseline measurement) and 36–38 weeks postpartum.<br>Participants reported their weight during a telephone interview at 10–12 weeks postpartum and 36–38 weeks postpartum. |
| Van Uytsel et al., 2022 | Wearable Activity Monitor (Withings Go), weighing scale (Withings Body+) & Mobile application | NA | The INTER-ACT intervention consisted of a combination of face-to-face lifestyle coaching and an e-health-supported smartphone application.<br>The face-to-face coaching sessions were conducted at 6 weeks, 8 weeks, 12 weeks, and 6 months postpartum, focusing on nutrition, physical activity, and mental well-being. In each session, motivational interviewing (Ask-Tell-Ask method, **transtheoretical model**), **behavior change techniques** (goal setting, action planning, self-monitoring, encouragement, etc.), and **shared decision-making** played a central role.<br>A Bluetooth connection was set up with an activity tracker (Withings Go) and a weighing scale (Withings Body+). The application facilitated self-monitoring, goal-setting and sent tailored motivational messages. | Weight change (kg) was calculated as the difference between 6 weeks postpartum (baseline measurement) and 6 months postpartum.<br>Weight was measured using a Tanita MC-780 SMA bioelectric impedance analysis (BIA) device. |
