# Supplementary material for: Effectiveness of the Internet of Things for Improving Pregnancy and Postpartum Women’s Health in High-Income Countries: A Systematic Review and Meta-Analysis of Randomized Controlled Trials
Source: Healthcare (Basel). 2025 Aug 23;13(17):2103. doi: 10.3390/healthcare13172103 (PMC12428080; doi:10.3390/healthcare13172103)
Supplement: Supplementary file 1 [file healthcare-13-02103-s001.zip › Table S8. Summary of outcomes reported in included studies, categorized as primary clinical outcomes and behavioralintermediate outcomes.pdf]

Table S8. Summary of outcomes reported in included studies, categorized as primary clinical outcomes and behavioral/intermediate outcomes.

Author, Year	Primary Clinical Outcomes*	Behavioral / Intermediate Outcomes**
Gilmore et al., 2017	None reported	Weight change (kg), Body fat (%), Waist circumference (cm)
Cheung et al., 2019	Postpartum OGTT attendance	Physical activity (self-reported and pedometer steps), Dietary macronutrient intake, Self-reported weight
Sung et al., 2019	Gestational age at delivery, Birth weight, Small/Large-for-gestational-age, Cesarean section	Maternal BMI, Weight, Body fat (%), HOMA-IR, Blood pressure, Fasting glucose
Chen et al., 2023	None reported	Excessive GWG rate (weekly and total), Total GWG, GWG trajectory
Gonzalez-Plaza et al., 2022	Unplanned cesarean, Composite pregnancy morbidity, Preeclampsia/gestational hypertension, Gestational diabetes, Miscarriage ≤22 weeks, Preterm labor ≤37 weeks, Birthweight ≤2,500 g, Perinatal death, Early neonatal death, Antepartum stillbirth	GWG (kg), Weekly GWG, Physical activity (moderate to high)
Lim et al., 2021	None reported	Postpartum weight change (achieving optimal weight), Calorie/activity goal attainment
Van Uytsel et al., 2022	None reported	Weight retention, Body fat (%), Waist and hip circumference, Physical activity (MET-min/week), Sedentary time, Energy intake

*Primary clinical outcomes include maternal and neonatal health outcomes directly related to clinical efficacy (e.g., obstetric complications, neonatal morbidity and mortality).

**Behavioral/intermediate outcomes include lifestyle or physiological measures that may act as mediators of clinical effects (e.g., physical activity, body composition, diet).
