# Supplementary figures and images for: Effectiveness of the Internet of Things for Improving Pregnancy and Postpartum Women’s Health in High-Income Countries: A Systematic Review and Meta-Analysis of Randomized Controlled Trials
Source: Healthcare (Basel). 2025 Aug 23;13(17):2103. doi: 10.3390/healthcare13172103 (PMC12428080; doi:10.3390/healthcare13172103)

Figure S1. Risk of bias graph.

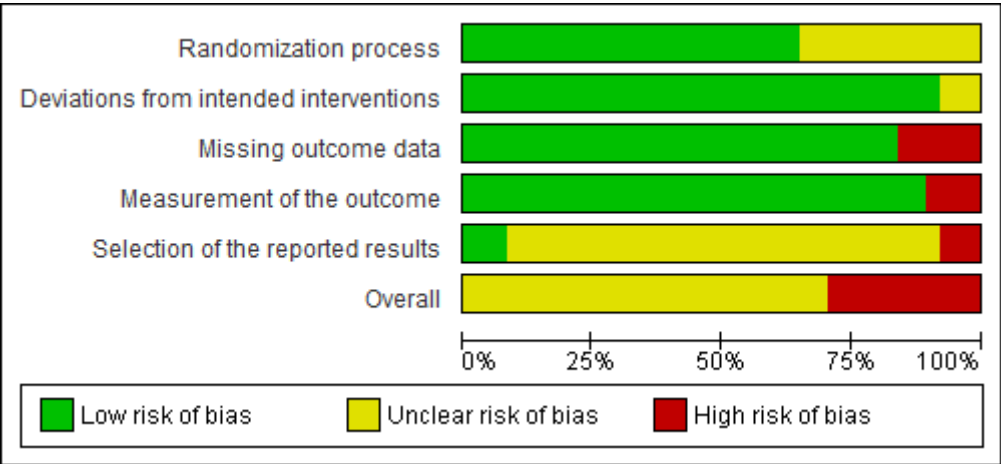

Supplement: Supplementary file 1 [file healthcare-13-02103-s001.zip › Figure S1. Risk of bias graph_rev.pdf]
